# Maternal chemosignals enhance infant-adult brain-to-brain synchrony

**DOI:** 10.1126/sciadv.abg6867

**Published:** 2021-12-10

**Authors:** Yaara Endevelt-Shapira, Amir Djalovski, Guillaume Dumas, Ruth Feldman

**Affiliations:** 1 Center for Developmental Social Neuroscience, The Interdisciplinary Center, Herzliya, Israel.; 2 Precision Psychiatry and Social Physiology Laboratory, CHU Sainte-Justine Research Center, Department of Psychiatry, Université de Montréal, Montreal, QC, Canada.; 3 Human Brain and Behavior Laboratory, Center for Complex Systems and Brain Sciences, Florida Atlantic University, Boca Raton, FL, USA.; 4 Yale University, Child Study Center, New Haven, CT 06519, USA.

## Abstract

Maternal body odors serve as important safety-promoting and social recognition signals, but their role in human brain maturation is largely unknown. Utilizing ecological paradigms and dual- electroencephalography recording, we examined the effects of maternal chemosignals on brain-to-brain synchrony during infant-mother and infant-stranger interactions with and without the presence of maternal body odors. Neural connectivity of right-to-right brain theta synchrony emerged across conditions, sensitizing key nodes of the infant’s social brain during its maturational period. Infant-mother interaction elicited greater brain-to-brain synchrony; however, maternal chemosignals attenuated this difference. Infants exhibited more social attention, positive arousal, and safety/approach behaviors in the maternal chemosignals condition, which augmented infant-stranger neural synchrony. Human mothers use interbrain mechanisms to tune the infant’s social brain, and chemosignals may sustain the transfer of infant sociality from the mother-infant bond to life within social groups.

## Teaser

Maternal chemosignals increase infant-adult interbrain synchrony, suggesting their role in social brain maturation.

## INTRODUCTION

For social mammals, a key function of the mother-infant bond is tuning the infant’s brain to life within the social ecology and building mechanisms for the detection of safety and danger (*1*, *2*). Primates, who require the help of allomothers to rear their slowly maturing young, must transfer this external regulatory function to other caring adults in the social group and imbue them with familiarity and safety (*3*–*5*). Odors are ancient, species-general signals that trigger complex neural changes that function to consolidate social bonds with conspecifics, augment the salience of contextual cues, and signify mother and habitat (*6*); odors are the only sensory cues that can represent the mother in her absence. While the role of olfaction in humans has received less attention compared with vision and audition, anthropological studies describe the reliance on odors for group living in non-Western societies, the recognition of group odor by its members, ceremonies by which a father’s smell is “transferred” to his infant, or the rubbing of body odors (BOs) by axilla sweat, suggesting that children integrate into social groups through the detection of familiar odors introduced to infants by their mother (*7*–*9*). Furthermore, studies have shown that human neonates rely on olfactory cues to recognize their mothers (*10*, *11*), and maternal chemosignals reduce pain in newborns (*12*), increase infant attention to face and eyes (*13*), shape face categorization (*14*), and attenuate neural response to fearful faces (*15*), underscoring the importance of maternal odors for orienting infants to species-critical social cues. Still, the mechanisms by which maternal BOs support maturation of the infant’s social brain are largely unknown. Detailing these processes may be important for elucidating the biological origins of human sociality.

A special feature of human social life is the key role ascribed to facial communication as the predominant mode of social contact. Human neonates preferentially attend to faces, particularly to eyes, and the salience of the facial channel renders face-to-face interactions the basic platform for the acquisition of social skills. Face-focused interbrain synchrony is a human-specific mechanism that uses humans’ inborn face preference to fine-tune the developing social brain. Research has shown that episodes of shared gaze enhance brain-to-brain coupling (*16*, *17*), and adults use the infant’s earliest social repertoire, such as visual attention to faces, laughter, and vocalizations, to synchronize with the child’s behavior and enhance interbrain coupling (*18*). Mother-infant interbrain synchrony may enhance the salience of social cues, increase infant social motivation, and function to externally regulate the infant’s immature brain and tune it to social living. It is thus possible that through chemosignals, mothers transfer this interbrain function to other adults in their surroundings to enable allomaternal care and expand infant socialization from the mother-infant bond to life within social groups.

In the current study, we tested the role of maternal BO in the development of interbrain synchrony between infants and unfamiliar adults to test the role of odors during a sensitive period for infant social brain maturation. Interpersonal synchrony describes the temporal linkage between the behavioral and/or physiological processes of social partners that may be concurrent or sequential (*19*), with the two forms of interbrain synchrony possibly reflecting distinct processes (*20*). Here, we examined infant-adult concurrent, nondirectional neural synchrony.

**Fig. 1.**
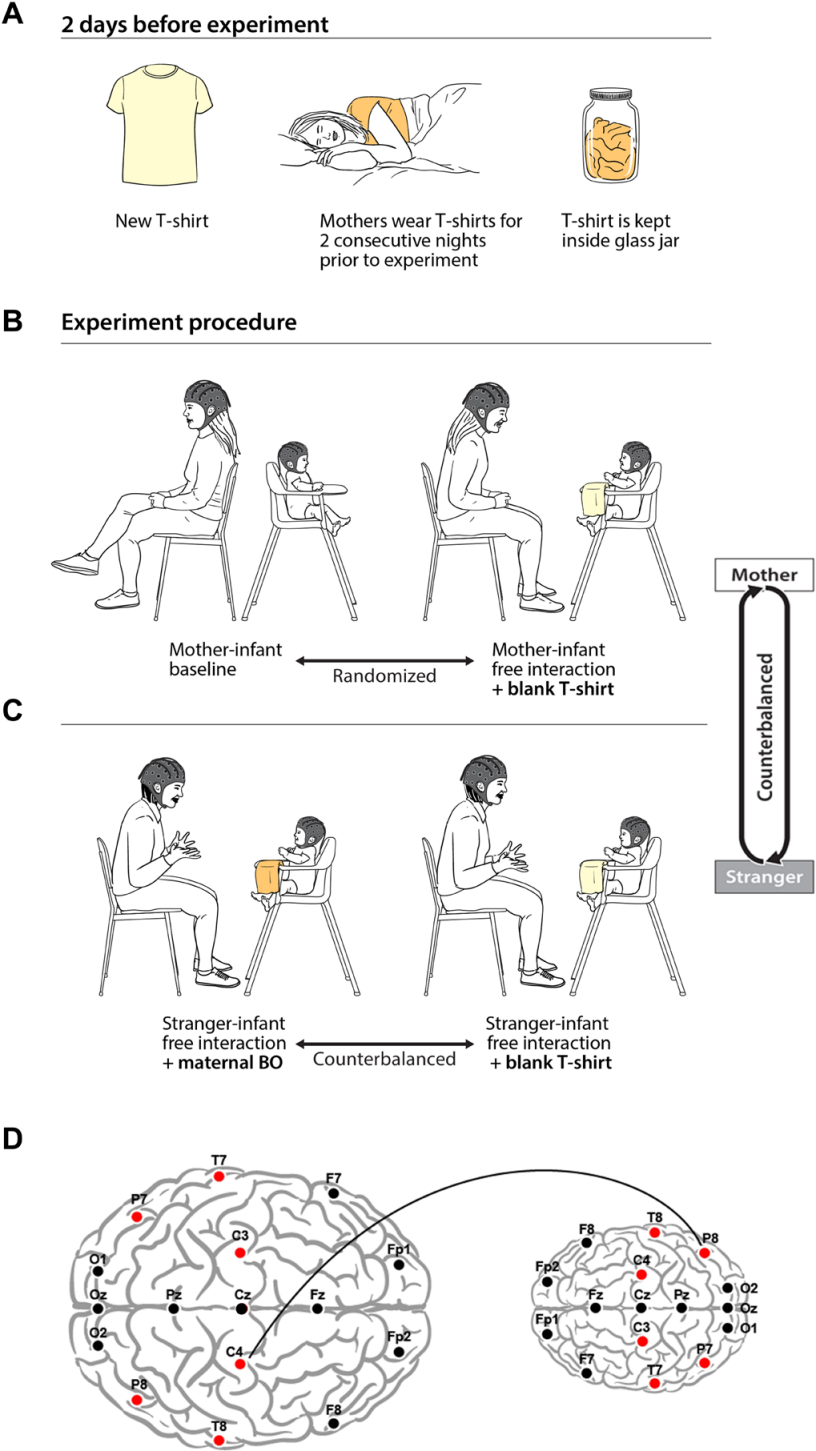
Experimental procedures. (**A**) BO collection. Two days before the experiment, mothers were given 100% cotton T-shirts to wear for two consecutive nights before the experiment. Between nights, the T-shirts were kept inside a closed glass jar and stored in the participants’ freezer. (**B**) Infant-mother paradigms. Infant and mother were fitted with EEG electrodes and participated in back-to-back paradigm and face-to-face free interaction paradigm. (**C**) Infant-stranger paradigms. Two consecutive interactions were conducted, once with continuous exposure to maternal BO and once in the presence of a new T-shirt placed in the same position. The two odor conditions were counterbalanced. (**D**) Illustration of infant-adult interbrain neural synchrony. Electrodes recorded and analyzed are marked in red dots; interbrain neural synchrony values were calculated for theta frequency band (4 to 7 Hz) using weighted phase lag index (wPLI). Connectivity scores were computed for all intersubject electrode (red dots) combinations, resulting in 36 wPLI values per dyad per condition. Connectivity between adult’s C4 and infant’s P8 is shown as example for interbrain neural connectivity.

We tested four hypotheses related to core processes underpinning infant-adult brain-to-brain synchrony. First, consistent with adult interbrain studies (*21*), we expected that mother-infant face-to-face interactions would elicit greater neural coupling compared with episodes when the two are in similar proximity but without facial and vocal communication (hypothesis 1, Fig. 1A). Second, neural processes that underpin attachment enable infants to recognize the mother and imbue her with salience, familiarity, and safety (*22*), and we thus expected greater interbrain synchrony between infants and their own mothers compared with a stranger (hypothesis 2, Fig. 1, B and C). Third, regarding the role of chemosignals in interbrain communication, we expected that the presence of maternal BO would attenuate the difference between infants’ neural synchrony with mother and unfamiliar female (hypothesis 3, Fig. 1C).

Our fourth hypothesis addressed potential moderators of the chemosignal effect during infant-stranger communication. We considered three behavioral processes. First, maternal chemosignals have been shown to increase infant visual attention to faces, particularly to eyes (*13*), and we expected that maternal BO would augment infant visual social attention. Second, maternal chemosignals are thought to enhance infant positive arousal (*13*), and infants may display more positive affect, laughing, and vocalizations in the BO condition (*18*). Last, studies in mammals indicate that maternal odor enhances safety and familiarity (*23*), which increase approach behavior and reduce social aversion, and we expected that in the BO condition, infants would show greater social approach and engagement. We thus examined whether the increase in interbrain coupling in the BO condition may be associated with the increase in visual attention, positive arousal, and social engagement observed in the presence of maternal odor (hypothesis 4). Overall, our goal was to shed light on the processes by which maternal chemosignals affect the human social brain during its early sensitive period and support infant socialization into group living.

## RESULTS

**Fig. 2.**
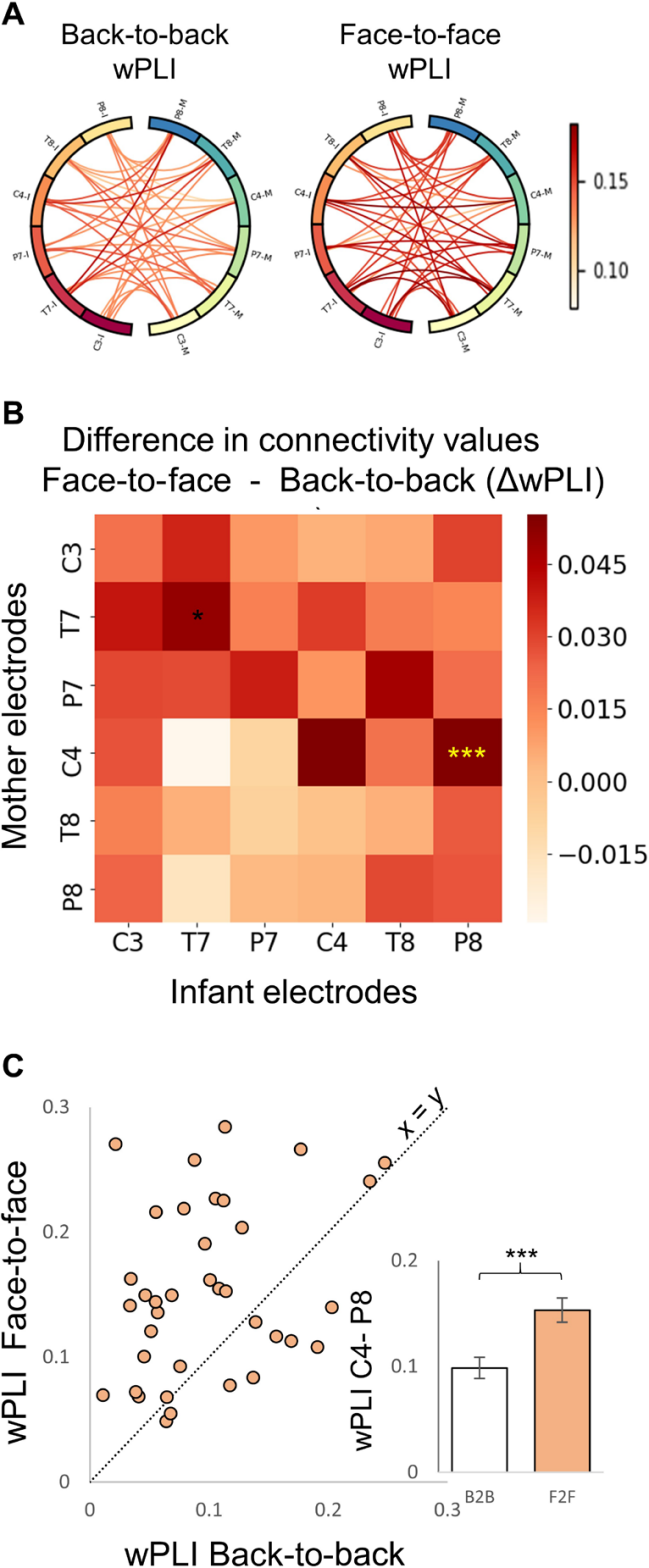
Higher interbrain neural synchrony during mother-infant face-to-face interaction compared with back-to-back condition. (**A**) Visualization of connectivity values (wPLI) during mother-infant back-to-back (B2B; left) and face-to-face (F2F; right) conditions. Each circle represents mean connectivity values for 36 combinations obtained from 37 infant-mother dyads. Within each circle, connections between infants’ electrodes (ends with “I”) and mothers’ electrodes (ends with “M”) are shown. (**B**) Difference in connectivity values across electrode combinations between the B2B and F2F conditions. The *x* axis represents the infant electrodes, and the *y* axis the mother electrodes. Dark red–colored squares represent comparisons with higher connectivity in the F2F condition compared with the B2B condition. Nonparametric permutation testing with mass-univariate ANOVA revealed significant main effect for condition (*F*_1,36_ = 19.3, *P* = 0.002). The significant comparison following permutation procedure is marked with yellow asterisks. (**C**) Interbrain neural synchrony between the right central area of the mother and the right occipitotemporal area of the infant. Each circle represents the connectivity score of a single participant following F2F (*y* axis) and B2B (*x* axis). The diagonal line reflects the unit slope line (*x* = *y*) such that if points accumulate above the line, then values are greater for F2F, and if they accumulate under the line, then values are greater for B2B. The bar graph represents the quantified results of the data shown in the scatter plot. ***P <0.001.

To test our first hypothesis, we used the connectivity scores obtained from 37 dyads that completed both the back-to-back and infant-mother free interaction paradigms consecutively and spent at least 60 s in the back-to-back paradigm (Fig. 2, A and B). Our primary analysis used nonparametric permutation test with mass-univariate analysis of variance (ANOVA) to detect effects associated with the face-to-face free interaction versus the back-to-back condition on the weighted phase lag index (wPLI) scores. The test was based on one-way repeated-measures ANOVA design. Results indicated significant main effect of experimental condition (face-to-face versus back-to-back; *F*_1,36_ = 19.3, *P* = 0.002, η^2^_p_ = 0.35). This effect indicated higher interbrain connectivity during mother-infant face-to-face interaction compared with the back-to-back condition between the right central area of mother and the right occipital-temporal area of infant (back-to-back: 0.10 ± 0.07, face-to-face: 0.15 ± 0.06) (Fig. 2C).

**Fig. 3.**
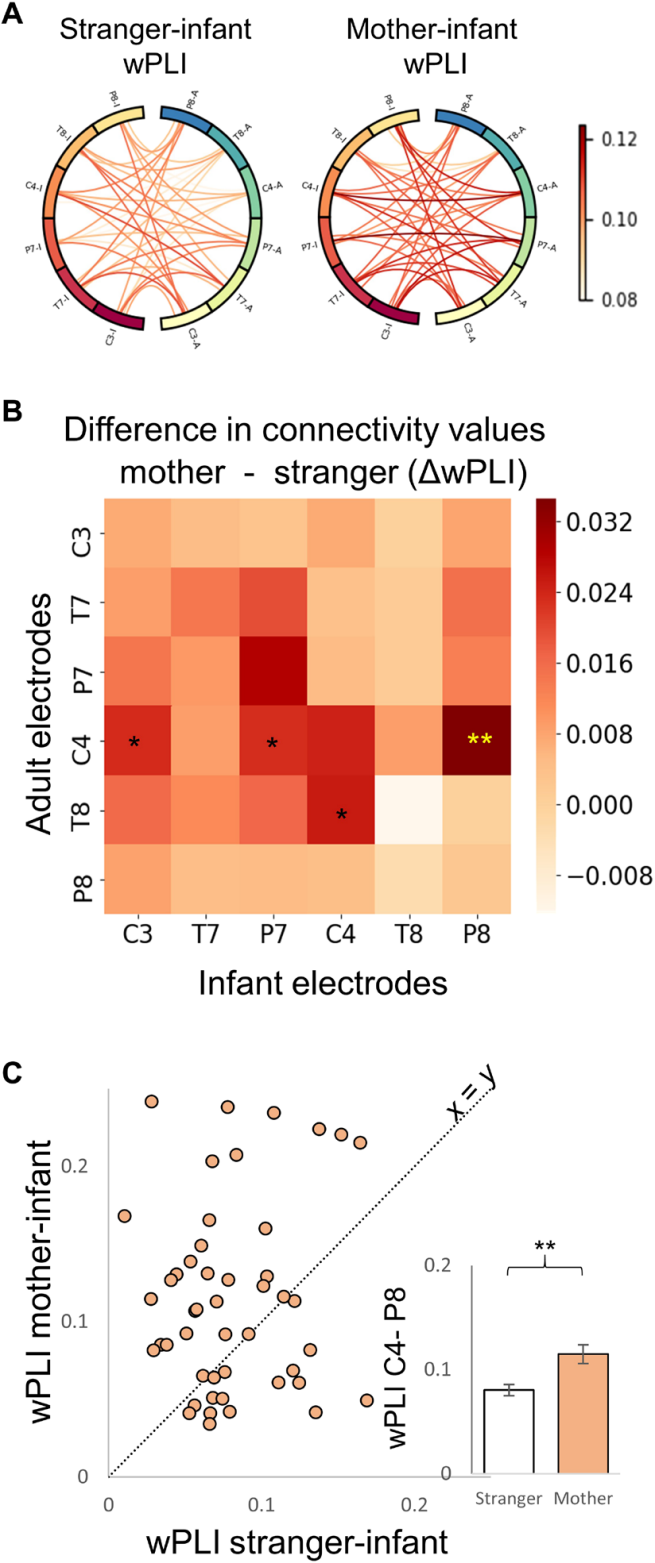
Higher interbrain neural synchrony during mother-infant compared with stranger-infant interaction. (**A**) Visualization of connectivity values in the infant-stranger (left) and the infant-mother free interaction (right) conditions. Each circle represents mean connectivity values for 36 combinations obtained from 47 infant-stranger dyads. Within each circle, connections between infants’ electrodes (ends with “I”) and adults’ electrodes (ends with “A”) are shown. (**B**) Difference in connectivity values across all electrode combinations between the infant-stranger and infant-mother conditions. The *x* axis represents the infant electrodes, and the *y* axis the adult electrodes. Nonparametric permutation test with mass-univariate ANOVA revealed a significant main effect of condition (*F*_1,46_ = 11.8, *P* = 0.026). The significant comparison following permutation procedure is marked with yellow asterisks. (**C**) Interbrain neural synchrony between the right central area of the adult and the right occipitotemporal area of the infant. Each circle represents the connectivity score of a single participant following mother-infant (*y* axis) and stranger-infant (*x* axis). The diagonal line reflects the unit slope line (*x* = *y*) such that if points accumulate above the line, then values are greater for mother-infant. The bar graph represents the quantified results of the data shown in the scatter plot. **P <0.01.

Next, we compared neural synchrony between infant-mother and infant-stranger dyads. The stranger was a woman of similar age as the participating mothers, had an infant at the same age range of the study infants, and lived in the same area, hence considered in-group member and potential allomother. The same stranger was used across participants. We used the wPLI connectivity scores obtained from 47 dyads who completed both mother-infant and stranger-infant (in the blank odor condition) paradigms (counterbalanced) (Fig. 3, A and B). Nonparametric permutation test with mass-univariate ANOVA revealed a significant main effect of interacting figure (mother versus stranger; *F*_1,46_ = 11.8, *P* = 0.026, η^2^_p_ = 0.20) [please see fig. S9 for the same analysis with phase locking values (PLV) (*24*)]. This effect indicated higher connectivity in the mother-infant condition compared with the stranger-infant condition between the right central area of the adult and the right occipitotemporal area of the infant (stranger: 0.08 ± 0.04, mother: 0.115 ± 0.06) (Fig. 3C).

### Exposure to maternal BO increases interbrain synchrony during infant-stranger social interaction

**Fig. 4.**
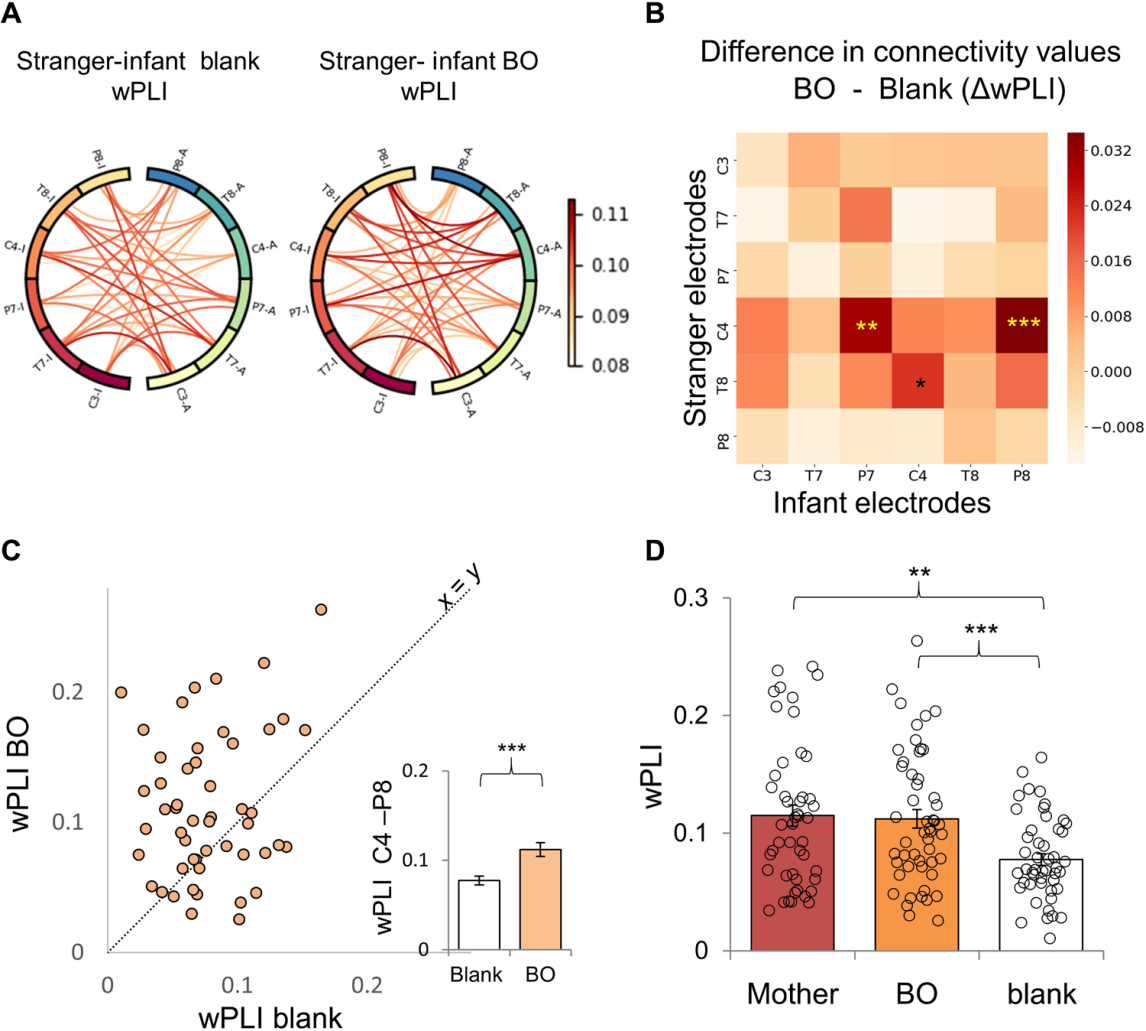
Increased interbrain neural synchrony following exposure to maternal body odor. (**A**) Visualization of connectivity values (wPLI) in the blank (left) and the BO (right) conditions. Each circle represents mean connectivity values for 36 combinations obtained from 51 infant-stranger dyads. Within each circle, connections between infants’ electrodes (ends with “I”) and stranger electrodes (ends with “A”) are shown. (**B**) Difference in connectivity values of all electrode combinations between the BO and blank condition. The *x* axis represents the infant electrodes, and the *y* axis the stranger electrodes. Nonparametric permutation test with mass-univariate ANOVA revealed a significant main effect of odor condition (*F*_1,50_ = 16.6, *P* = 0.005). The significant comparisons following permutation procedure are marked with yellow asterisks. (**C**) Interbrain neural synchrony between the right central area of the stranger and the right occipitotemporal area of the infant. Each circle represents the connectivity score of a single participant following BO (*y* axis) and blank (*x* axis). The diagonal line reflects the unit slope line (*x* = *y*) such that if points accumulate above the line, then values are greater for BO compared with blank. The bar graph represents the quantified results of the data shown in the scatter plot. (**D**) Interbrain neural synchrony between the right central area of the adult and the right occipitotemporal area of the infant in three conditions: mother-infant, stranger-infant with maternal body odor, and stranger-infant in blank condition. **P <0.01, ***P <0.001.

Our findings show that interactions between infants and their own mothers are characterized by significantly higher interbrain neural synchrony compared with interactions between infants and a female stranger. These differences are particularly noted over the connection between the adult’s right central area and the infant’s right occipitotemporal connection. Following these finding, we set out to explore whether exposure to maternal BO collected from the mothers (Fig. 1A) would facilitate behavioral and neural synchrony between infants and the female stranger during social interaction. We measured synchrony between infant and stranger during face-to-face free interaction in two conditions: once in the presence of maternal BO and once in a control condition (blank odor), and conditions were counterbalanced (Fig. 1C). We used the wPLI connectivity scores obtained from 51 infants (mean age, 6.8 ± 1.3 months) who completed the interactions in both odor conditions (counterbalanced) (Fig. 4, A and B). Our primary analysis used a nonparametric permutation test with mass-univariate ANOVA to find effects associated with the odor (maternal BO versus a control odor) on the interbrain synchrony during social interaction with stranger. Results revealed a significant main effect of odor condition (*F*_1,50_ = 16.6, *P* = 0.005, η^2^_p_ = 0.25) (please see fig. S10 for the same analysis with PLV values). This effect indicated higher connectivity in BO condition relative to blank between the right central area of the stranger and the right occipitotemporal area of the infant (BO: 0.11 ± 0.06, blank: 0.08 ± 0.04) (Fig. 4C) and also between the right central area of the stranger and the left occipitotemporal area of the infant (fig. S1). To further explore the effects of maternal BO on neural connectivity between infants and adults, we also compared directly the connectivity values between the BO condition and the mother-infant interaction. This comparison revealed no significant differences in connectivity (all *F* < 6.3, all corrected *P* > 0.39). Specifically, we compared the neural synchrony scores of the right central area of the stranger and the right occipitotemporal area of the infant between the mother and BO conditions. This comparison revealed no difference between mother and BO conditions in neural synchrony scores (*t*_46_ = 0.07, *P* = 0.94) (Fig. 4D).

### Maternal BO increases infants’ visual attention, safety and engagement, and positive arousal

Last, to test hypothesis 4, we examined the effects of maternal BO on infant social behavior: visual attention, safety and engagement, and positive arousal. To this end, each paradigm was coded offline using two well-validated coding schemes: the social behavior global coding scheme Coding Interactive Behavior (CIB) and microlevel second-by-second coding scheme. Social gaze and affect, the main nonverbal channels of social communication, were microcoded.

#### Infant visual attention

**Fig. 5.**
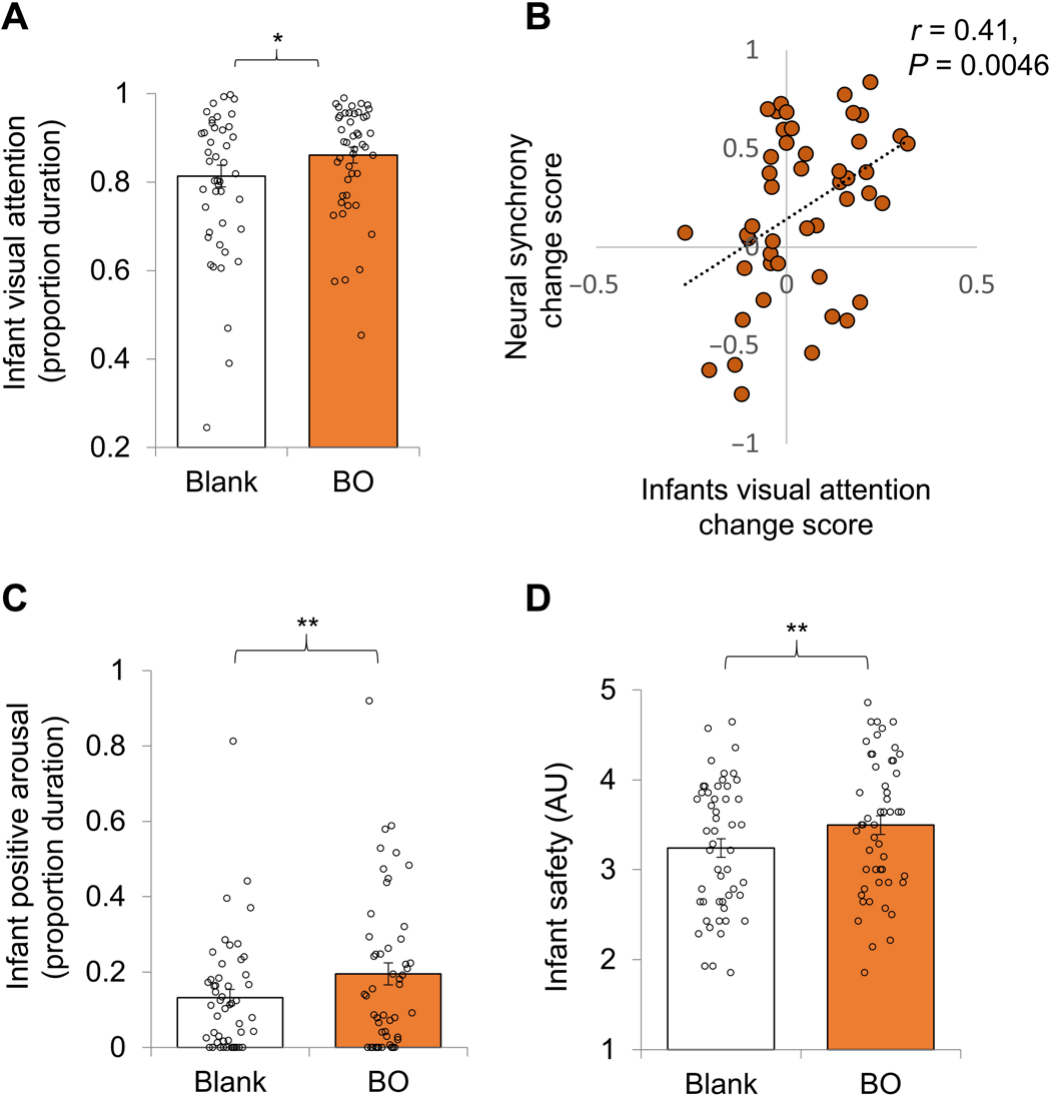
Increased visual attention, positive arousal, and feeling of safety following exposure to maternal BO. (**A**) Means ± SEM of infants’ visual attention proportion duration (*N* = 49) obtained in two odor conditions: blank (white) and BO (orange). We found higher infant visual attention in the BO condition compared with the blank condition. (**B**) The relation between neural synchrony change score of each dyad and infants gaze change score (*N* = 47, *r* = 0.41, *P* < 0.01). (**C**) Means ± SEM of infants’ positive arousal proportion duration (*n* = 50) obtained in two odor conditions: blank (white) and BO (orange). We found higher infant positive arousal in the BO condition compared with the blank condition. (**D**) Means ± SEM of CIB infant safety scores (*n* = 51) obtained in two odor conditions: blank (white) and BO (orange). We found higher infant safety in the BO condition compared with the blank condition. *P <0.05, **P <0.01.

Infant social gaze is a key nonverbal social behavior undergoing rapid maturation in the second 6 months. Significant difference emerged between conditions, indicating higher infant visual attention toward stranger in the BO condition compared with the blank condition (BO: 0.86 ± 0.13, blank: 0.81 ± 0.17, *t*_48_ = 2.2, *P* = 0.035; Fig. 5A). To test whether social attention mediated the elevation in interbrain synchrony in the BO condition, we tested whether the extent of maternal BO effect on neural synchrony was related to the extent of visual attention by correlating the neural synchrony change scores with the visual attention change scores from the blank to the BO condition. Interbrain neural synchrony change score correlated with the visual attention change score (*N* = 47, *r* = 0.41, *P* = 0.0046; Fig. 5B), such that greater increase in visual attention following exposure to BO is associated with greater increase in neural synchrony.

#### Positive arousal

Next, we tested the effects of BO on infant’s positive arousal, laughter, and positive vocalization obtained from the microcoding. Infant positive arousal was higher in the BO compared with blank condition (BO = 0.20 ± 0.20, blank = 0.13 ± 0.15, *t*_49_ = 2.7, *P* = 0.009; Fig. 5C). However, the degree of increase in positive arousal in the BO condition was not associated with the change in neural synchrony (*r* = 0.006, *P* = 0.96).

#### Safety and engagement

Using the CIB global scores, we analyzed effects of maternal BO on infants’ sense of safety, approach, and social engagement. Infant approach and engagement increased in BO compared with the blank condition (BO = 3.5 ± 0.75, blank = 3.24 ± 0.73, *t*_50_ = 2.9, *P* = 0.0053; Fig. 5D). However, the degree of increase in infant engagement in the BO condition was not associated with the change in neural synchrony (*r* = 0.15, *P* = 0.29).

## DISCUSSION

The socialization of infants to life within social communities requires the integration of multisensory signals of mother and habitat—among which are olfactory cues that promote safety, mark the in-group, and enable approach (*6*, *7*)—with neural processes, such as brain-to-brain synchrony. Interbrain processes use humans’ inborn face preference as a potential gateway for brain maturation. Our study charts a human-specific trajectory by which infant socialization expands from the mother-infant bond to life within social groups through the integration of maternal chemosignals and face-to-face interbrain synchrony. While research on interbrain communication has recently gained empirical attention as an emerging perspective on the socially embedded brain (*25*, *26*), ours is the first study to integrate BOs with the assessment of neural synchrony during a sensitive period for infant brain maturation. Furthermore, the fact that our main results were replicated by two methods of interbrain connectivity speaks to the robustness of the chemosignal effect and its replicability across neural computational methods.

Several important processes are highlighted by the findings that may play a role in human sociality. First, direct mother-infant face-to-face communication that includes shared gaze and vocalizations elicited greater interbrain synchrony as compared with moments of similar mother-infant proximity that did not include facial or vocal communication. It appears that already in their first social exchanges, infants imbue direct facial-vocal exchanges with importance and salience. This is consistent with extant developmental literature indicating that moments of mother-infant face-to-face interaction carry lasting effects on the developing brain and foster the consolidation of complex social behavior, such as focused attention, emotion regulation, social collaboration, and empathy (*27*, *28*). The potential for neural coupling may be one process that underpins the long-term and pervasive effects of face-to-face communication in infancy.

Second, neural coupling was tighter between infants and their own mothers compared with an unfamiliar female, consistent with a recent functional near-infrared spectroscopy (fNIRS) hyperscanning study of older children with mothers and strangers (*29*). While the exact reasons for this enhanced synchrony are not fully clear, several mechanisms, such as genetic similarity, greater maternal attunement, or familiarity with the partner’s rhythms beginning in utero that lead to increased infant engagement, may be at work. Future studies may tease apart these components by comparing mother-infant neural synchrony with father-infant or adoptive parent-infant coupling as well as by focusing on sequential synchrony to pinpoint adult versus child influences on interbrain processes (*30*).

Third, we describe the role of maternal chemosignals in leveling out the difference between neural coupling with mother and stranger, thus enabling the survival-critical function of allomothering. The presence of maternal BO increased the infant’s odor-sensitive social behavior, particularly visual attention to faces, positive arousal, and approach behavior. Possibly, the production of these social behaviors in the infant may have led to greater infant-adult behavioral synchrony, which, in turn, linked with greater neural coupling. The embeddedness of neural coordination within moments of visual and affective synchrony has been shown between long-term couples, but not among strangers (*31*), and our findings may describe a similar linkage between an infant and a stranger in the presence of the familiar maternal odor, but not when these odors are absent. Overall, our findings demonstrate the effects of maternal chemosignals not on the single brain but on two-brain coordination, a capacity theorized to have played a key role in the evolution of the human brain (*25*, *32*).

Across conditions and interacting partners, adult-infant brain-to-brain synchrony implicated the early-maturing right brain (*33*–*35*) and rode on theta rhythms that characterize the developing brain (*36*–*38*). Furthermore, across paradigms and comparisons, we detected a specific interbrain connection—between the right central area of the adult and the right occipitotemporal area of the infant—charting right-to-right centrotemporal theta connectivity. This specific connectivity has been shown in another hyperscanning electroencephalography (EEG) study to underpin adult imitation and was found between the model’s right central and the imitator’s right temporal regions in the same electrodes (*39*). During face-to-face interactions, mothers and infants engage in imitation games (e.g., peek-a-boo games), and imitation is critical for the development of social cognition, self-other differentiation, theory of mind, and body schema (*40*). Possibly, this right-to-right centrotemporal connectivity, which sustains the early interactions between infant and mother, also underpins human imitation throughout life and its important embodied function.

While exact localization of neural regions is not possible with EEG, and future research using other methods is required to pinpoint the brain areas detected here with greater specificity, our results repeatedly highlight connectivity with the infant’s occipitotemporal region, an area implicated in core social functions. The right occipitotemporal cortex comprises several brain regions, including the fusiform gyrus (*41*) and the superior temporal sulcus (STS), a key node of the social brain implicated in simulation, mentalization, and action observation (*42*). Consistent with our findings, neural synchrony between mothers and preadolescents has been detected in the right STS during the observation of own, but not unfamiliar interaction (*43*) in a magnetoencephalography study. Similarly, the effect of maternal BO on infant occipitotemporal region is consistent with prior research showing response in occipitotemporal areas to BOs. For instance, maternal BO was found to increase infants’ face categorization, particularly over the right occipitotemporal cortex (*14*). Adults’ response to BOs measured by positron emission tomography showed increased regional cerebral blood flow in the right anterior occipitotemporal cortex following exposure to a friend’s BO, which correlated with the duration of the friendship (*44*). Similarly, two adult functional magnetic resonance imaging studies indicated that the right fusiform gyrus responds to human sweat and differentiates anxiety-related and sexual arousal–related sweat (*45*, *46*). It thus appears that the right occipitotemporal cortex functions to identify olfactory social cues and integrate them into social contexts. While caution is needed with regard to the definition of specific brain areas, our findings may suggest that odor-sensitive areas modulate neurobehavioral dynamics in infants to potentiate interbrain synchronization. This may function to tune infants to in-group social partners, familiar social contexts, and the distinct components of social experiences.

Neural synchrony connected regions in the right hemisphere in both the adult’s and infant’s brains. The right hemisphere in humans and other mammals sustains functions critical for survival, such as visuospatial attention, interpretation of social information, and emotional processes (*47*). The “right hemisphere hypothesis,” proposed nearly 50 years ago (*48*), posits a general dominance of the right hemisphere for all emotions and is supported by studies showing dominance of the right hemisphere for multiple emotional functions (*49*). Because of its critical role in survival-related functions and nonverbal communication, right hemisphere dominance is thought to have appeared early in animal evolution and to mature early in human ontogeny (*34*); right hemisphere dominance is found at birth (*50*) and persists throughout the first 3 years of life (*33*, *35*).

Consistent with this early right hemisphere dominance model (*48*), a recent study assessed structural maturation from 3 to 12 months and found greater age-related cerebral blood flow increases in the right hemisphere. Structural maturation began in the primary sensorimotor cortex at 3 to 6 months and continued with posterior-temporal and frontal regions at 7 to 12 months, similar to the age range of our infants (*35*). This maturation pattern, from sensorimotor to temporal and prefrontal regions, is consistent with other studies utilizing different methods (*51*, *52*). The later maturation of the posterior-temporal region suggests that human-specific social experiences during this sensitive period, such as face-to-face play and imitation games observed universally, may define critical environmental inputs for maturation of this brain region. Our results, which consistently show adult neural synchrony with the infant’s posterior-temporal region across partners and conditions, may suggest that neural linkage with this area embedded within real-life social moments may contribute to its maturation during the sensitive period, but this hypothesis requires much further research.

Infant-adult synchrony with both mother and stranger implicated theta rhythms. This is consistent with a prior hyperscanning EEG study of infants and adults that described synchrony of theta and alpha rhythms (*16*). To verify that our findings are specific to theta oscillations, we conducted control analysis over all frequency bands, confirming that the right-centro-right-occipito-temporal synchrony found here is specific to theta rhythms (see figs. S2 to S4). Theta rhythms are characteristic of the developing brain (*38*, *53*), and this may be one reason for the theta synchrony found here. In addition, theta-band interbrain synchrony was found to increase during cooperation paradigms in adults (*54*–*57*) and during direct gaze in infant-adult exchanges, similar to our findings (*16*). In both infants and adults, among the multiple functions of theta rhythms is their role in the processing of emotional cues. In adults, recognition of distinct emotions is associated with theta synchronization in the right parietal cortex, with theta rhythms over right-central regions sustaining emotional processing (*58*). In infants, theta activity has been linked with basic life functions; increased theta is associated with feeding and basic positive and negative stimuli (*59*, *60*) and implicate occipitotemporal regions and right hemisphere dominance (*61*). Theta increases in bilateral posterior temporal regions during sucking and crying, suggesting that these rhythmic, life-sustaining functions arise from the posterior temporal cortex driven by neuronal impulses from the limbic system (*60*). However, since theta rhythms are implicated not only in emotional processes but also in a host of cognitive, attentive, and physiological processes, the reasons for theta-band synchrony in infant-adult hyperscanning research should be further investigated.

Three behavioral processes were hypothesized to mediate the effects of maternal BO on infant-stranger neural synchrony—visual attention to face, positive arousal, and safety/approach. While significant increases emerged in all three behaviors in the presence of maternal BO, only the increase in visual attention was directly linked with the increase in infant-stranger interbrain theta coupling. This suggests a possible mechanistic link between infants’ social attention and neural synchrony, which should be investigated in future research. This mechanism is consistent with interbrain studies in infants, which showed stronger bidirectional Granger-causal influences during direct compared with indirect gaze (*16*), and in adults, which indicated higher gamma power correlation during moments of social gaze (*31*). Although these two studies used different forms of synchrony (sequential and concurrent) that may reflect distinct phenomena (*20*), both demonstrated a causal role for social gaze in neural coupling. The greater social attention and positive arousal caused by the familiar odor may have increased dyadic investment and set the stage for tighter coupling, consistent with adult studies showing greater neural coupling during moments of positive effect embedded in face-to-face interactions (*18*). We suggest that maternal BO increases infants’ attention to ongoing social stimuli, such as eye gaze, facial expressions, laughter, and gestures, and these, in turn, induce theta-phase resetting. In parallel, infants’ increased production of social signals, such as laughter and vocalizations, leads to theta-phase resetting in the adult, culminating in mutual-phase resetting (*16*) (*62*) and greater interbrain synchrony between the infant and unfamiliar members of the social community. However, it is important to emphasize that infant hyperscanning research is a field in its infancy, and much further research is needed to elucidate the mechanisms that underpin change in infant-adult neural coupling, the factors that underpin this phase resetting, and the conditions that may enhance or impede neural coupling between infants and adults.

Across species, maternal odors enhance safety and familiarity, relax vigilance, and trigger neural processes by which the young form a memory trace of mother and habitat (*23*). In humans, we similarly found that maternal BO increased infants’ sense of safety, enhanced approach, and reduced social aversion. These findings suggest that maternal presence functions as a “safety signal” for human infants, allowing them to allocate less resources to danger signals and focus on social engagement and emotional processing (*15*). Possibly, in humans, the species-general function of safety integrates with the effects of odor on positive arousal and visual attention to allow infants to safely explore their social environment and neutrally connect with its members.

Several study limitations should be acknowledged. First, each mother has a unique BO signature that carries information related to genetic makeup (*63*), personal and environmental factors, and information about her emotional state (*64*), and we cannot pinpoint the specific components in the maternal BO responsible for the observed effects. We could speculate that breastmilk odor components may have played a role in the observed effects; however, we had a small group of nonbreastfed infants who still showed similar neural effects compared with the breastfed infants (*U* = 182, *Z* = 1.5, *P* = 0.13). Furthermore, since maternal BO was compared to a blank shirt, it is possible that the effects are colored by general infant arousal and are not specific to maternal BO or to the measured social factors. Still, our infants were constantly exposed to other human odors, including the stranger’s, and the environment was far from sterile, further supporting the observed effects. Our study represents the first effort to test odor effects on neural synchrony, and future research should compare odors of different sources, both human and nonhuman, that include pleasant and unpleasant odors.

Second, we tested the effects of maternal BO on neural synchrony between infants and unfamiliar mother of the same social group. Thus, while we do not hypothesize that the effects are specific to in-group mothers, future studies with male and female nonparents, fathers, and females from a different culture are needed for the generalization of the findings.

Last, infant-adult hyperscanning research is an emerging field of neuroscience, and thus, caution is needed in the interpretation of the findings. Potentially, the observed connectivity values may have been driven by spurious phenomena. To further verify that our results reflect true effects and are not driven by artifacts, we compared our connectivity values to shuffled data. This analysis revealed true right-centro-right-occipito-temporal interbrain synchrony during mother-infant face-to-face interaction and stranger-infant interaction in the presence of maternal BO (fig. S5). Another challenge in infant-adult hyperscanning studies is that neural oscillations in infants are slower than their functional equivalents in adults. Future research should thus explore this issue by comparing classical single-frequency coupling with alternative cross-frequency coupling techniques (*65*). Along with this, in the current study, we decided to use wPLI as a measure for interbrain synchrony to avoid spurious hyperconnections that could result from similar sensory experiences of the participants, and our main results are also replicated using PLV. However, there are other methods to measure interbrain connectivity, each with its own drawbacks and benefits.

In summary, a series of ecological paradigms and dual EEG recording converge to describe a specific interbrain neural connectivity of right-to-right brain theta synchrony. We suggest that this specific connectivity plays a key role in human social brain maturation during a sensitive period of its development. We show that maternal presence experienced only through her smell increased infants’ visual attention to social signals, augmented positive arousal, and improved safety during interaction with a stranger, enhancing interbrain synchrony in this specific connectivity. Infants require their mother’s presence for growth, soothing, and survival, but the two must be copresent at the same place and time for this presence to be felt. The unique properties of olfaction, which preserves maternal presence in her absence, can assist infants in transitioning to social groups, exploring new environments, and communicating with unfamiliar partners, augmenting their social repertoire toward greater survival and thriving. Much further human research is required to understand the role of olfaction in development, its impact on social brain maturation, and the mechanisms that enable two humans to create a coupled biology toward greater social connection.

## MATERIALS AND METHODS

The study was preregistered—https://osf.io/avzme/?view_only=c5f7ef9197ca48e8a60d49581007ad2d.

### Participants

All procedures used here, including paradigms, questionnaires, and equipment, were approved by the Interdisciplinary Center Institutional Review Board committee. All the mothers who participated in the experiment with their infants signed an informed consent. All procedures were explained to the mothers before the study and were performed in accordance with ethical guidelines. Participants were free to leave the experiment at any time with full compensation. The participants were recruited through online forums and social media groups.

The following were the inclusion criteria to participate in the experiment: the mother was not pregnant at the time of the experiment, the infant must be the mother’s biological child, both the infant and the mother are healthy at the time of the experiment, and both the mother and the infant are not diagnosed with epilepsy.

Overall, 150 participants (75 mothers and 75 infants) arrived at the laboratory. Yet, different numbers of participants completed each paradigm. Eight infants completed none of the paradigms, and two infants were excluded from the experiment because they were sick when they arrived at the laboratory. The remaining were 62 infant-mother dyads who completed the free interaction paradigm [mother age, 33.3 ± 4.0 years; infants, 28 female (F) and 34 male (M), mean age, 7.0 ± 1.49 months], 39 dyads who completed the bubbles paradigm (mother age, 32.7 ± 4.3 years; infants, 16 F and 23 M, mean age, 6.8 ± 1.1 months), and 51 infant-stranger dyads who completed both the BO and the blank conditions (stranger age, 34 years; infants, 25 F and 26 M, mean age, 6.8 ± 1.3 months) (table S1). Because of excessive noise, EEG data of one dyad in the mother-infant free interaction paradigm were excluded.

### Procedure

#### BO collection

The donors (mothers) were provided with 100% cotton T-shirts. The donors were instructed to wear the shirt for two consecutive nights before the experiment day. The donors were asked not to use soap, shampoo, conditioner, or deodorant before they wear the shirt. Between the two nights, the T-shirts were kept inside a closed glass jar that were stored in the donors’ home freezer.

#### Paradigm

Both the infant and the adult (the mother and the stranger mother) were fitted with 17 EEG electrodes. During the experiment, the infants were sitting in a high baby chair or in a baby bouncer chair, depending on their ability to sit by themselves. All paradigms were videotaped for later offline coding. The experiment included the following paradigms:

##### Mother-infant back-to-back paradigm

Both the infant and the mother were watching soap bubble machine simultaneously while they were sitting back-to-back, using two separate bubble machines. The mothers were instructed to avoid interaction with their infant. In this specific paradigm, the infants were less cooperative and had difficulties to remain calm. We excluded data where infants were crying or turned around and tried to have contact with their mother during the paradigm. We included only infants that were sitting calmly for at least 60 s, remaining with 38 infants for the connectivity analysis. We restricted the duration for analysis for the first 90 s. Following connectivity analysis, we excluded a single dyad whose connectivity values exceeded more than 3 SDs of the group mean.

##### Mother-infant free face-to-face interaction paradigm

The infant and the mother were left alone in the experiment room. The mothers were instructed to avoid physical contact with the infants and avoid using other objects. The duration of the interaction was approximately 3 min.

##### Stranger-infant free face-to-face interaction paradigm

The same stranger participated in all experiments; she is also a mother, and at the time of the experiment, her age ranged between 34 and 35 years. The infant and the stranger were left alone in the experiment room. Two consecutive interactions were conducted: once during constant exposure to the BO of the infant’s own mother and once in the presence of a new T-shirt, which is the blank condition. The two odor conditions were counterbalanced. The T-shirts (BO/blank) were placed under the infants’ neck in the cases where they were sitting in the baby bouncer chair or on the feeding tray of the high chair.

The adult stranger avoided physical contact with the infants and avoided using other objects. The duration of the interactions was approximately 3 min each.

#### Randomization and blinding

Odor conditions were counterbalanced in order across participants. The experiment was double blinded, i.e., both participants, infants and the stranger mother, were blind to experimental conditions. In addition, the order of the infant-mother and the infant-stranger interaction was randomized.

#### Dual-EEG data acquisition

Neuroelectric activity in both participants of each dyad was simultaneously and continuously recorded using Brain Products GmbH. The system was composed of two Acticap helmets with 16 active electrodes arranged according to the international 10/20 system. The reference was fixed on fronto-central electrode, FCz. The impedances were maintained below 10 kilohms. Both subjects were connected to the same amplifier that guaranteed millisecond-range synchrony between the two EEG recordings.

#### EEG preprocessing

The preprocessing was conducted using Python 3.6.6 in Anaconda (v4.7.12) with MNE software suite (v0.17.0). First, for the preprocessing procedure, we separated the EEG data file of each dyad to infant data file and adult data file. Then, we applied a 1- to 50-Hz band-pass filter. Next, following segmentation of the signal to 1-s epochs with 500-ms overlap between epochs, we applied an automatic algorithm that detects noisy segments. Similar to other hyperscaning EEG research (*26*), we used the MNE “AutoReject” v0.1 algorithm (*66*) with Bayesian optimization as the threshold method. AutoReject is an automatic data-driven algorithm for detection and repair of bad segments using optimal peak-to-peak rejection thresholds subject-wise. In the BO condition, 10.8 ± 13.7% of the epochs were rejected; in the blank condition, 8.7 ± 14.2% of the epochs were rejected; in the mother-infant free interaction condition, 13.7 ± 16.7% of the epochs were rejected; and in the mother-infant back-to-back condition, 13.7 ± 16.9% of the epochs were rejected. To verify this method, randomly selected segments were visually inspected for excessive noise in the EEG signal. The AutoReject algorithm removes trials containing transient jumps in isolated channels but does not necessarily work well for a systematic physiological artifact that affects multiple sensors. For these purposes, we used MNE’s implementations of FastICA and CORRMAP (*67*). CORRMAP allows manual selection of an independent component (IC) for exclusion in one participant and use the chosen component as a template for selecting and excluding similar components in other participants. The general idea behind the CORRMAP algorithm is that artifact patters are generally similar over a large number of participants. Therefore, correlation between the template IC and each IC solution enables choosing the IC with the highest correlation. Thus, excluding similar components, we identified and removed components containing ocular movements. Overall, in the BO paradigm, mean of 1.67 ± 1.04 components per subject were detected and removed; similarly, in blank condition, mean of 1.69 ± 1.04 components were detected and removed. In the mother-infant face-to-face interaction, mean of 1.65 ± 1.24 components were detected and removed, and in the back-to-back paradigm, mean of 1.54 ± 1.04 components per subject were detected and removed.

To compare the intersubject connectivity scores between two conditions (BO versus blank, mother-infant versus stranger (blank), and mother-infant interaction versus back-to-back), the duration of each dyad in both conditions was matched by taking the minimal duration of both conditions for each dyad. Following EEG preprocessing, the epochs of both subjects (adult and infant) in each paradigm were matched such that only data points with “good” epochs for both subjects are included. Overall, there was no significant difference in the final epoch number between conditions (BO = 237 ± 64 (20% rejection), blank = 243.8 ± 64.3 (17% rejection), *t*_50_ = 0.8, *P* = 0.4; mother-infant free interaction = 217.5 ± 67.7 (25% rejection), stranger (blank) = 237.6 ± 60.6 (18% rejection), *t*_46_ = 1.5, *P* = 0.13; mother-infant free interaction = 117.4 ± 39.8 (26% rejection), back-to-back = 122.4 ± 33.6 (24% rejection), *t*_36_ = 0.54, *P* = 0.59).

#### Connectivity analysis

The adult-infant interbrain neural connectivity values were calculated for theta frequency band (4 to 7 Hz). We focus on theta frequency band because theta oscillations are strongly related to the processing of emotional cues and behavioral states with substantial attentional and emotional load. In infants, social stimulation, periods of sustained attention, and exploration of novel objects are associated with increased theta oscillations (*36*). In our current study, similar to Dikker *et al.* (*68*), we wanted to avoid spurious hyperconnections that could result from similar sensory experiences of the participants, which are not related to the social interaction itself. Despite the subliminal presentation of the olfactory stimuli (embedded within the T-shirt), we wanted to avoid “false-positive” connectivity results from potential perceptual differences between blank and BO conditions, as the exposure to similar input may result in increased synchrony, which is not related to the social interaction between the infant and the stranger. Moreover, in our back-to-back condition, both the infant and the mother were watching soap bubbles machine simultaneously. These shared visual stimuli may also induce zero-lag synchronization. To avoid spurious hyperconnections that could result from similar sensory experiences of the participants, we decided to use wPLI as our measure for interbrain synchrony. wPLI is an extension of the PLI. By weighing each phase difference according to the magnitude of the lag, phase differences around zero only marginally contribute to the calculation of the wPLI. This procedure reduces the probability of detecting false-positive connectivity in the case of noise sources (in our case, shared environmental inputs at the perceptual level as described above) with near zero phase lag and increases the sensitivity in detecting phase synchronization. (*69*). The wPLI is a robust and widely used method for magnetoencephalography (MEG)/EEG functional connectivity. wPLI ranges between 0 and 1, where 0 indicates no synchrony and 1 indicates full synchrony.

Consistent with previous research (*16*, *17*, *26*, *31*, *43*) and to avoid a large number of multiple comparisons, we choose to focus on interbrain synchrony only in temporal (T7, T8), occipital-temporal (P7, P8), and central (C3, C4) regions. These regions were previously indicated to be involved in interbrain neural synchrony, particularly in studies using more ecological settings. Using hyperscanning EEG, neural synchrony in central regions was found between infant and adult when singing nursery rhymes, which increased during episodes of live direct gaze compared with previously recorded video (*16*). A similar dual-EEG study found synchrony in temporal regions between romantic couples during natural positive conversation (*31*). An fNIRS study showed that eye-to-eye condition, compared with eye-to-picture condition, increased activity in the left superior and temporal gyri as well as in pre- and supplementary motor cortices (*17*). A MEG study showed synchrony in the right STS between mothers and children while observing their own interaction in the home ecology but not when observing unfamiliar interaction (*43*).

#### Statistical analysis

The neural connectivity statistical analysis was performed with eelbrain, an open source Python module for accessible statistical analysis of MEG and EEG data (v0.31.7, https://github.com/christianbrodbeck/eelbrain, DOI 10.5281/zenodo.598150). All connectivity values for each pair of electrodes for each subject and condition entered the analysis.

For analyzing the differences between each of the two conditions (BO versus blank, mother versus stranger, and face-to-face versus back-to-back) and to avoid multiple comparisons, we used nonparametric permutation test with mass-univariate ANOVA (*70*). We used a permutation test since it uses a distribution derived from permuting the observed scores rather than assuming that the population has a specific distribution, for instance, a normal distribution (*71*).

Our main hypotheses in the current study, which uses a within-subjects design, are related to comparison between conditions and not between electrode pairs within a single condition. Thus, we used repeated-measures ANOVA to compute the *F* value for each of the electrode pairs, comparing between conditions within dyads (subjects). The same procedure was repeated in 1000 random permutations of the original data, shuffling condition labels within subject to take into account the within-subject nature of the design. For each permutation, the largest *F* value was retained to form a nonparametric estimate of the distribution of the largest *F* value under the null hypothesis that condition labels are exchangeable. Last, a *P* value was computed for each electrode pair in the original *F*-map as the proportion of permutations that yielded a comparison with a larger *F* value than the comparison under question. Only electrode pairs, which reached a *P* value of 0.05 or smaller following permutation procedure, are reported.

### Behavioral analysis—The CIB

To assess behavioral measures, each paradigm was coded offline using both microcoding analysis and social behavior coding scheme [CIB; (*72*)] The CIB coding system is a global measure that looks at parent, infant, and dyadic affective states and interactive styles. The CIB uses a variety of social settings and observational paradigms (e.g., free play and feeding) and has been used in studies in healthy and high-risk populations and clinical trials. The CIB is broken down into parent, infant, and dyadic codes that are rated on 5-point Likert scales. Infants Safety score is composed of the following parameters: adult supportive presence, infant positive affect, infant initiation, infant lead interaction, dyadic reciprocity, dyadic adaptation-regulation, and dyadic tension (the last parameter was reordered to be in the same direction as the other parameters). The scores were computed for all dyads in both stranger-infant conditions: in the control odor condition and in the maternal BO condition. Coding was conducted by two coders blind to odor condition who were trained to 85% reliability on all codes. Interrater reliability, computed for 15% of the interactions, averaged 93% (intraclass *r* = 0.94).

The second coding scheme we used was a microlevel second-by-second coding scheme previously validated in our laboratory (*73*). Social gaze and affect, the main nonverbal channels of social communication were coded using a computerized system [Mangold Interact, Mangold International GmbH (Ed.), www.mangold-international.com]. Specifically, we were interested in infants’ social gaze and positive affect as these two have previously been linked to interbrain neural synchrony (*31*). Interrater reliability for 15% of the tapes was 92% (kappa = 0.89).

The following codes were used for each infant:

1. Infant gaze:

a. Gaze to parent/social gaze – Infant gazes socially at the adult’s face.

b. Gaze to object – Infant is looking at object other than the object of joint attention.

c. Joint attention – Infant and adult are looking at the same object.

d. Gaze aversion – Infant gazes away from the adult but is not focused on other objects or the environment. Usually, facial expression and muscle tone are loose.

e. Gaze to environment – Infant’s gaze is scanning the environment (gaze is not focused on objects, people, or other elements of the environment).

f. Drowsy/tired – Meant to indicate any signs of drowsiness or fatigue.

g. Uncodable gaze – Indicating that because of filming or other limitations, it is impossible to code this category.

2. Infant effect:

a. Positive effect – Infant expresses clear signs of joy and exuberance with overt smiles or laughter.

b. Neutral effect – Infant is content, and facial expression is bright but no signs of high positive arousal.

c. Negative effect – Infant is either withdrawn or in negative arousal, including crying, fussing, whining, etc.

d. Uncodable effect – Indicating that because of filming or other limitations, it is impossible to code this category.

For the visual attention measurement, we calculated the exact proportion duration that infants gazed toward the adult, including moments of joint attention and excluding moments of crying and fussing. For the positive affect, we calculated the exact proportion duration that infants were in a positive state, including smiling and laughing. We excluded one participant since most of his interaction was uncodable due to technical issues, and one participant was excluded from the “visual attention” analysis since there was disagreement between coders with regard to his gaze parameters.

To test for a possible correlation between behavioral measurements and interbrain neural synchrony, we calculated a change score for each participant in both measurements: the difference between odor conditions divided by the maximum value of both conditions: $\frac{\left(\text{BO}-\text{Blank}\right)}{\text{max}\;(\text{BO},\text{Blank})}$. We excluded two participants whose ratio between conditions exceeded more than 3 SDs from the group mean.

## Notes

View/request a protocol for this paper from *Bio-protocol*.
