# Supplementary material for: Maternal chemosignals enhance infant-adult brain-to-brain synchrony
Source: Sci Adv. 2021 Dec 10;7(50):eabg6867. doi: 10.1126/sciadv.abg6867 (PMC8664266; doi:10.1126/sciadv.abg6867)
Supplement: Supplementary file 1 — Figs. S1 to S11 Table S1 [file sciadv.abg6867_sm.pdf]

Science Advances
AAAS

Supplementary Materials for

Maternal chemosignals enhance infant-adult brain-to-brain synchrony

Yaara Endevelt-Shapira*, Amir Djalovski, Guillaume Dumas, Ruth Feldman*

*Corresponding author. Email: yaara.yaara@gmail.com (Y.E.-S.); feldman.ruth@gmail.com (R.F.)

Published 10 December 2021, *Sci. Adv.* **7**, eabg6867 (2021)
DOI: 10.1126/sciadv.abg6867

This PDF file includes:

Figs. S1 to S11
Table S1

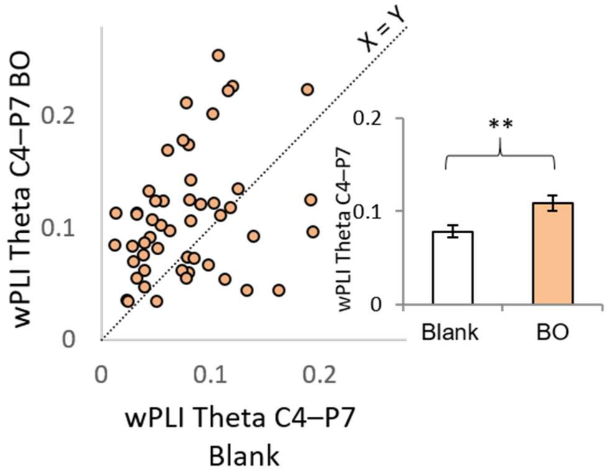

Fig. S1.

Interbrain neural synchrony between the right central area of the stranger and the left occipito -temporal area of the infant (n = 51 dyads). Each circle represents the connectivity score of a single participant following BO (y axis) and control (x axis). The diagonal line reflects the unit slope line (x=y) such that if points accumulate above the line then values are greater for BO and if they accumulate under the line then values are greater for control. The bar graph represents the quantified results of the data shown in the scatter plot. Non - parametric permutation test revealed a significant main effect of Odor condition ($F(1,50) = 13.5$, corrected $p = 0.02$) This effect indicated higher connectivity in BO condition relative to Blank between the right central electrode of the Stranger (C4) and the left occipito-temporal electrode of the infant (P7) (BO: 0.11 ± 0.06, Blank: 0.08 ± 0.05)

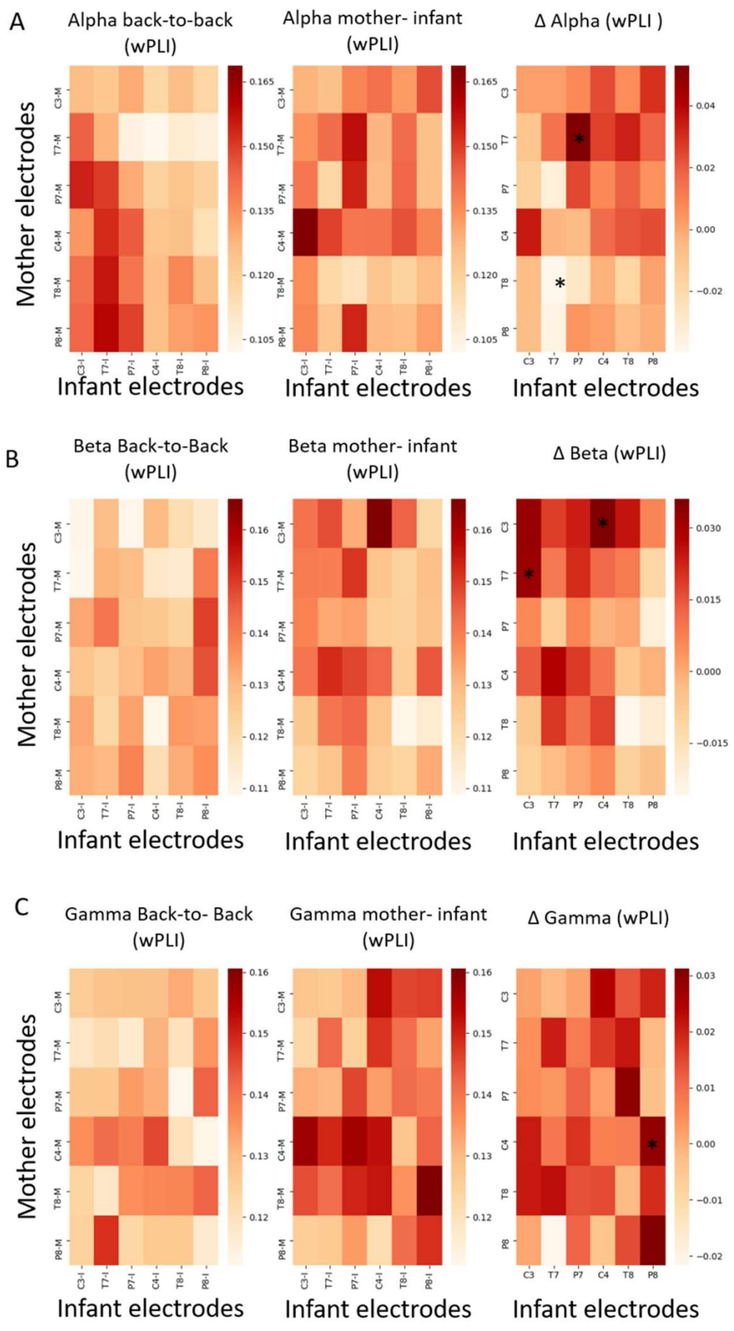

Fig. S2.

Connectivity comparison between face-to-face mother-infant interaction and back-to-back in (A)alpha (8-12 Hz), (B) beta (13-20 Hz) and (C) Gamma (32- 40 Hz) frequency bands. The heatmaps represents the mean connectivity values for 36 combinations obtained from 37 infant-mother dyads in the back-to-back condition (left), mother-infant free interaction condition (middle) and the difference between conditions (right) . The x-axis represents the Infant electrodes and the y-axis the mother electrodes. Dark red colored squares represents comparisons with higher connectivity in the free interaction condition compared with back-to-back , while light yellow represents comparisons with higher connectivity in the back-to-back condition compared with free interaction. Significant comparisons (before correction for multiple comparisons) are marked with asterisks. Results indicated no significant main effects for all frequency bands (all $F < 6.0$, all corrected $p > 0.4$).

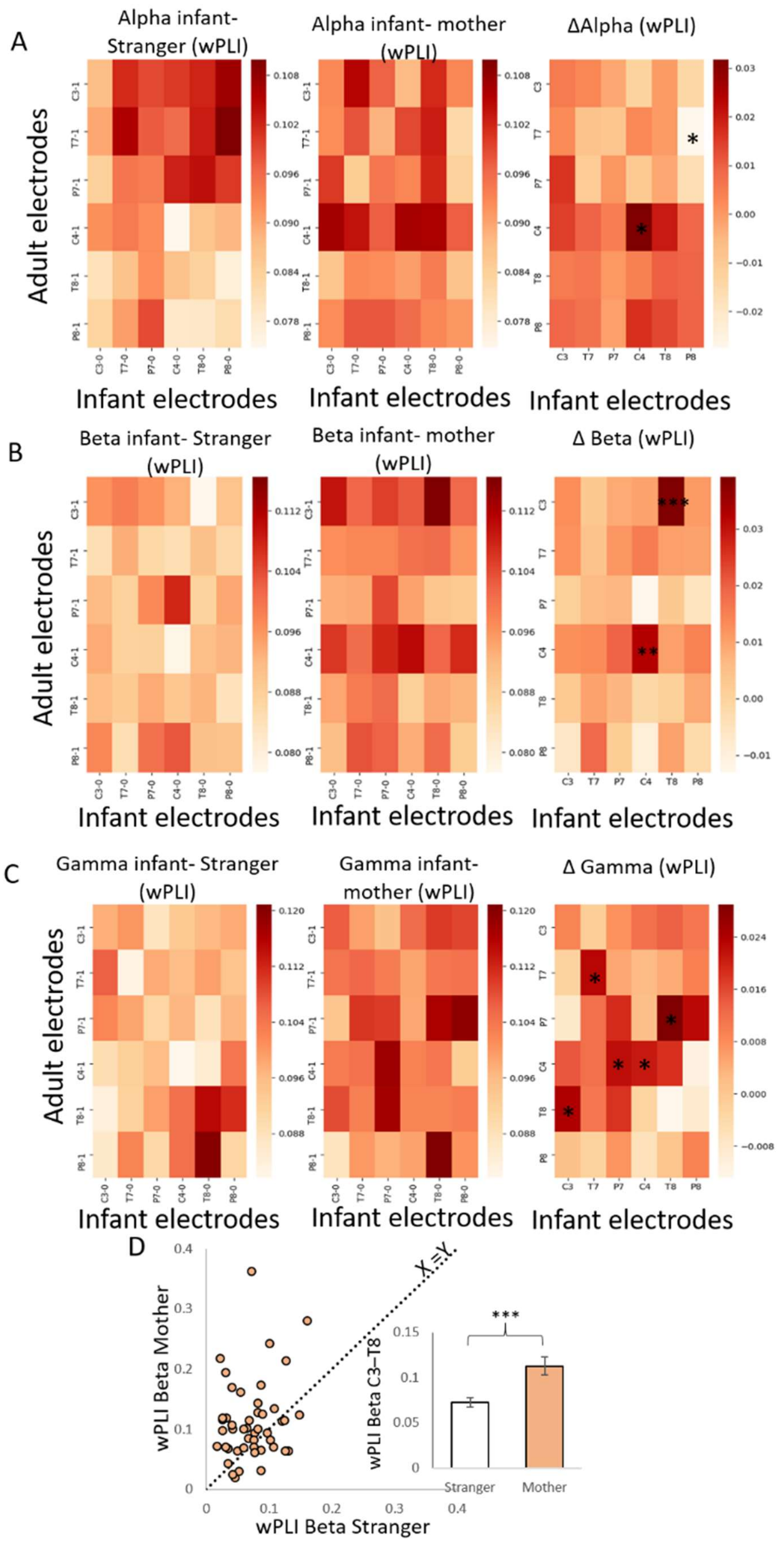

A
Alpha infant-Stranger (wPLI)
Alpha infant- mother (wPLI)
ΔAlpha (wPLI)
Adult electrodes
Infant electrodes
B
Beta infant- Stranger (wPLI)
Beta infant- mother (wPLI)
Δ Beta (wPLI)
C
Gamma infant- Stranger (wPLI)
Gamma infant- mother (wPLI)
Δ Gamma (wPLI)
D
wPLI Beta Mother
wPLI Beta Stranger
X=Y
wPLI Beta C3–T8
***
Stranger
Mother

Fig S3.

Connectivity comparison between face-to-face mother-infant interaction and stranger- infant interaction in (A)alpha (8-12 Hz), (B) beta (13-20 Hz) and (C) Gamma (32- 40 Hz) frequency bands. The heatmaps represents the mean connectivity values for 36 combinations obtained from 47 infant-mother dyads in the stranger-infant condition (left), mother-infant free interaction condition (middle) and the difference between conditions (right) . The x-axis represents the Infant electrodes and the y-axis the adult electrodes. Dark red colored squares represents comparisons with higher connectivity in the mother condition compared with stranger, while light yellow represents comparisons with higher connectivity in the stranger condition compared with mother-infant free interaction. Significant comparisons (before correction for multiple comparisons) are marked with asterisks. (D) The permutation test revealed significant main effect of interacting figure in beta ($F = 13.3$, corrected $p = 0.024$), yet no main effects were found for either alpha or gamma (all $F < 6.6$, all corrected $p > 0.38$). The effect indicated higher connectivity in the mother-infant condition compared with the stranger-infant condition between adult C3 - infant T8 (Stranger: 0.08 ± 0.04, Mother: 0.11 ± 0.06)

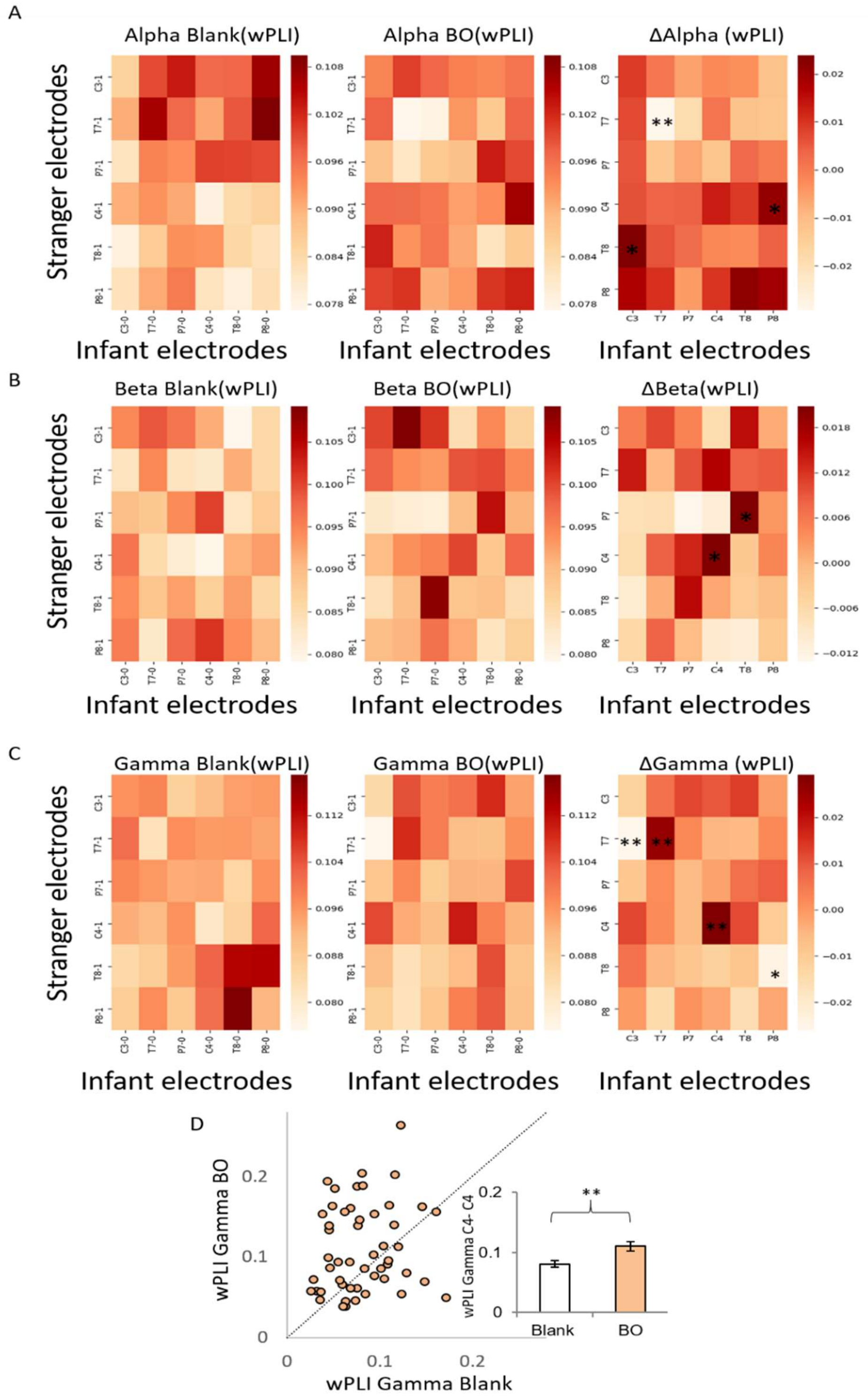
A
Alpha Blank(wPLI)
Alpha BO(wPLI)
ΔAlpha (wPLI)
Stranger electrodes
Infant electrodes
C3-1 T7-1 P7-1 C4-1 T8-1 P8-1
C3-0 T7-0 P7-0 C4-0 T8-0 P8-0
C3 T7 P7 C4 T8 P8
0.108 0.102 0.096 0.090 0.084 0.078
0.02 0.01 0.00 −0.01 −0.02
B
Beta Blank(wPLI)
Beta BO(wPLI)
ΔBeta(wPLI)
Stranger electrodes
Infant electrodes
0.105 0.100 0.095 0.090 0.085 0.080
0.018 0.012 0.006 0.000 −0.006 −0.012
C
Gamma Blank(wPLI)
Gamma BO(wPLI)
ΔGamma (wPLI)
Stranger electrodes
Infant electrodes
0.112 0.104 0.096 0.088 0.080
0.02 0.01 0.00 −0.01 −0.02
D
wPLI Gamma BO
wPLI Gamma Blank
0 0.1 0.2
wPLI Gamma C4- C4
Blank
BO
**

Fig S4.

Connectivity comparison between odor conditions in (A) alpha (8-12 Hz), (B) beta (13-20 Hz) and (C) Gamma (32-40 Hz) frequency bands. The heatmaps represents the mean connectivity values for 36 combinations obtained from 51 infant-stranger dyads in the Blank condition (left), Maternal BO condition (middle) and the difference between conditions (right) . The x-axis represents the Infant electrodes and the y-axis the adult electrodes. Dark red colored squares represents comparisons with higher connectivity in the BO condition compared with Blank while light yellow represents comparisons with higher connectivity in the Blank condition compared with BO. Significant comparisons (before correction for multiple comparisons) are marked with asterisks. (D) The permutation test revealed significant main effect of odor in gamma Stranger C4- Infant C4 (BO: 0.11 ± 0.05, Blank: 0.08 ± 0.04, $F = 11.8$, corrected $p = 0.031$), yet no main effects were found for either alpha or beta (all $F < 6.5$, all corrected $p > 0.35$)

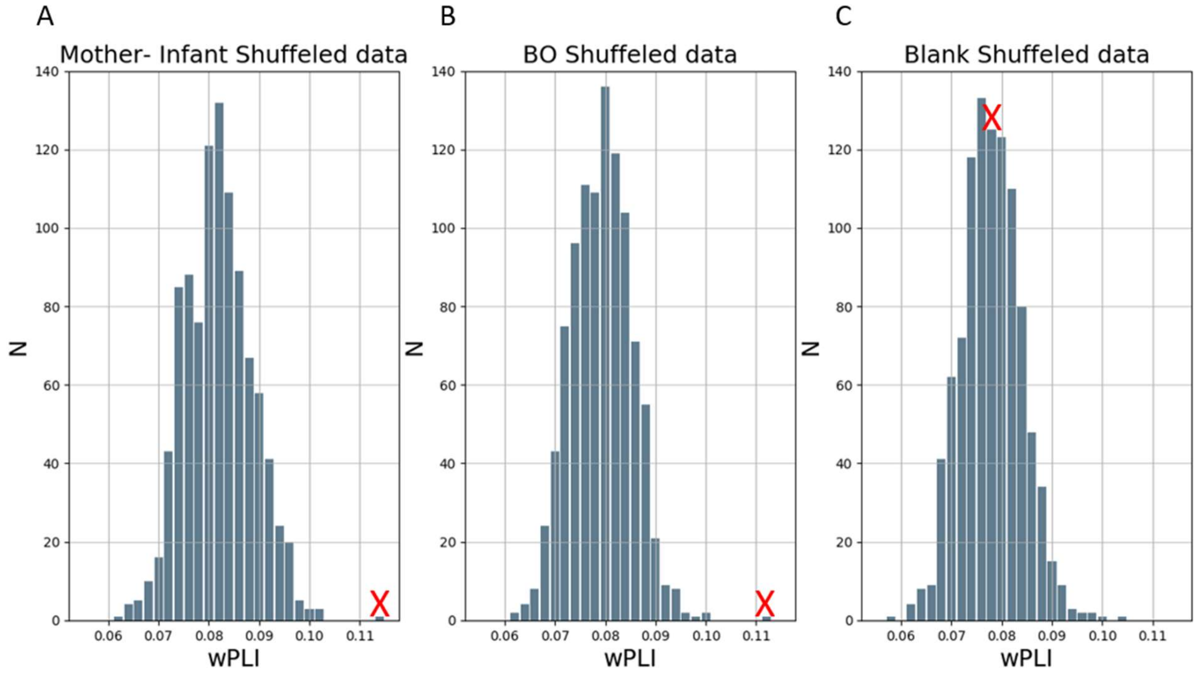

Fig S5.

Histogram of Mean Shuffled wPLI scores for (A) mother-infant (B) stranger- infant with maternal body-odor and (C) stranger- infant with Blank t-shirt. The red 'X' represents the real mean of wPLI scores. For each of the paradigms separately, we randomly shuffled the epochs of one member in each dyad 1000 times and compared the original connectivity values of the right central- right occipitotemporal connection with the connectivity values obtained from the shuffled data of the right central- right occipitotemporal connection. For the mother-infant face-to-face interaction, 98 % of the comparisons the mean of the original data was significantly higher (one-sample t-test, $p < 0.05$) compared with the shuffled data (A). Similarly, the infant- stranger BO condition, in 99% of the comparisons the mean of the original data was significantly higher (one-sample t-test, $p < 0.05$) compared with the shuffled data (B). However, no difference was found between the original data of the Blank condition and its shuffled data (C) and not for the back-to-back condition and its shuffled data. This analysis reveals that while no right central- right occipitotemporal interbrain synchrony exists during the stranger-infant control odor interaction nor during the back-to-back condition, there is a real genuine right central- right occipitotemporal interbrain synchrony during mother-infant face-to-face interaction and both during stranger- infant interaction in the presence of maternal body odors. Moreover, there is no difference in the shuffled data mean scores (averaging the 1000 sample for each dyad) between the three face-to-face paradigms; mother-infant 0.82 ± 0.022 (A), stranger-infant BO 0.79 ± 0.016 (B), stranger-infant Blank 0.78 ± 0.015 (C) (BO vs Blank, $t(50) = 0.6$, $p = 0.56$, Blank stranger-infant vs mother-infant, $t(46) = 0.89$, $p = 0.38$). This analysis was conducted in order to verify that the observed inter- personal neural synchrony is differentiated from spurious synchrony that could be driven by common intrinsic properties of the signal or consistent external perturbation during the experiment

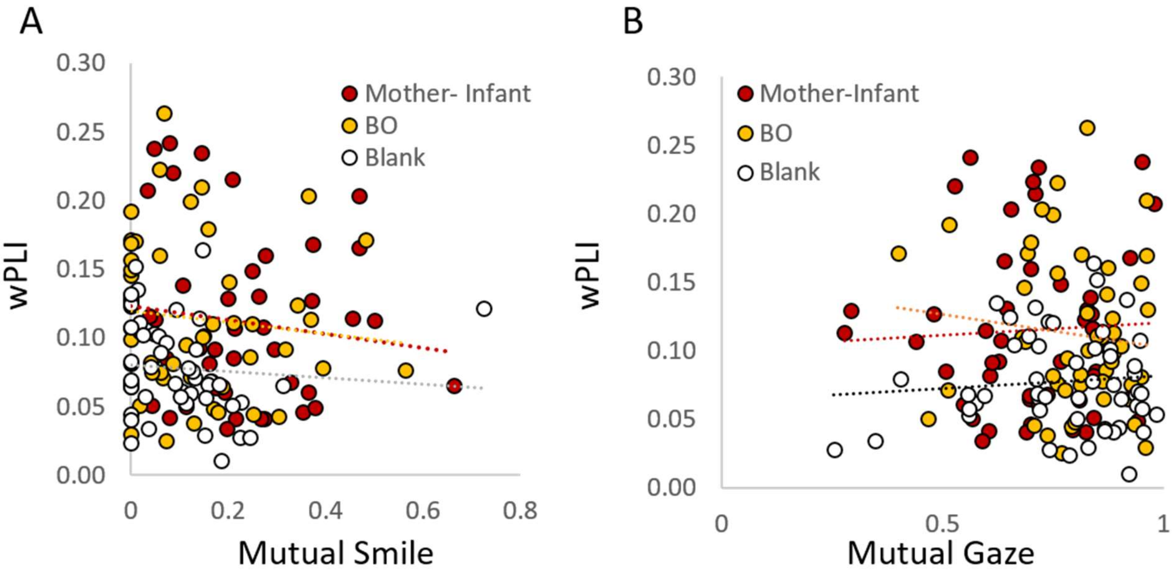

Fig S6.

The relation between Behavioral and neural scores. A. The relation between neural synchrony scores and mutual smile scores for: Mother-infant paradigm (red) Stranger-infant with BO (yellow) and stranger- infant with Blank t-shirt (white). We found no correlation between the synchronized behavior and the specific neural synchrony scores in all three paradigms (Mother-infant: $r = -0.13$, $p = 0.38$, BO: $r = -0.1$, $p = 0.49$, Blank: $r = -0.08$, $p = 0.57$) B. The relation between mutual gaze scores and the right central- right occipito-temporal connectivity scores. We found no correlation between the synchronized behavior and the specific neural synchrony scores in all three paradigms (Mother-infant: $r = 0.05$, $p = 0.76$, BO: $r = -0.11$, $p = 0.45$, Blank: $r = 0.09$, $p = 0.55$).

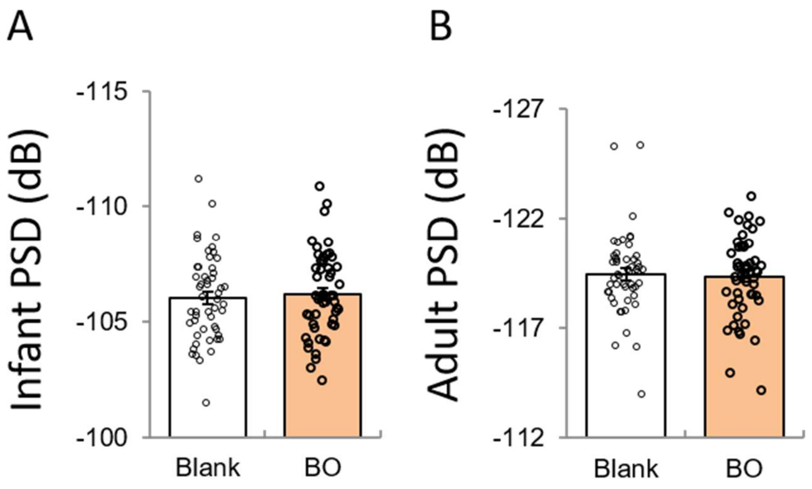

Fig S7.

No difference in Power between odor conditions.

To validate that the observed differences in wPLI are not related to changes in power following the added odor stimuli, theta power spectral density (PSD) was calculated on the right central area of the adult and the right occipito-temporal area of the infant for both odor conditions. We used the MNE's implementation of PSD calculation using multitapers. We compared PSD scores between BO and Blank conditions for infants and adults, separately. The analysis revealed no significant difference in power between the BO and the Blank condition for both (A) infants' electrode ($t(50) = 1.7$, $p = 0.09$, Cohen's $d = 0.1$) and (B) adults' electrode comparison ($t(50) = 0.43$, $p = 0.67$, Cohen's $d = 0.05$).

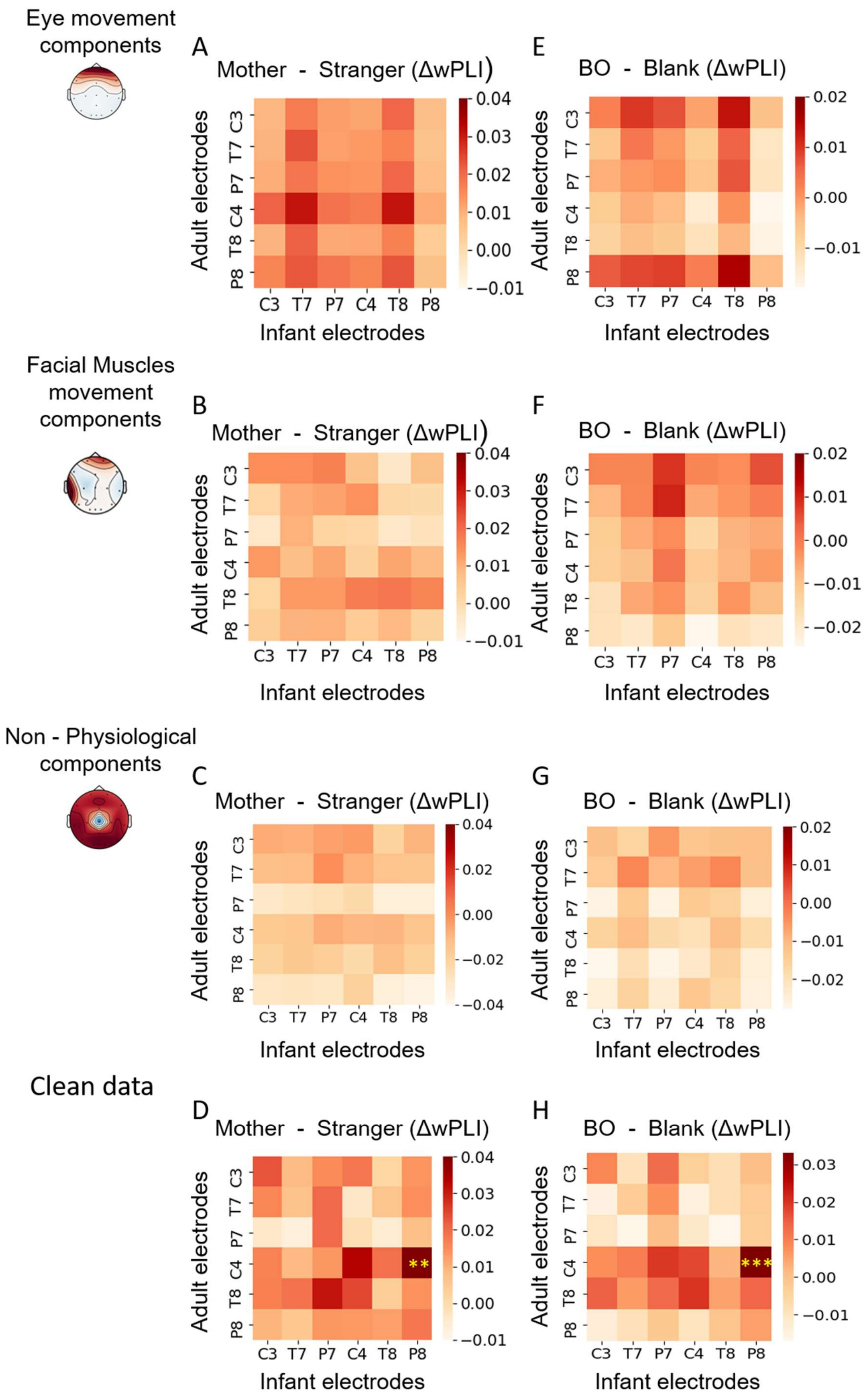
Eye movement components
A
Mother - Stranger (ΔwPLI)
E
BO - Blank (ΔwPLI)
Facial Muscles movement components
B
Mother - Stranger (ΔwPLI)
F
BO - Blank (ΔwPLI)
Non - Physiological components
C
Mother - Stranger (ΔwPLI)
G
BO - Blank (ΔwPLI)
Clean data
D
Mother - Stranger (ΔwPLI)
H
BO - Blank (ΔwPLI)
Adult electrodes
Infant electrodes
C3 T7 P7 C4 T8 P8
**
***

Fig S8.

Exploring the possibility that artefactual components are contributing to the effects of interest

In order to explore the possibility that artefactual components are contributing to the effects of interest, we identified and differentiated between facial muscles components, eye movement components and non – physiological components. The identification was based on topographies and power spectrum. Importantly, the selected templates were balanced across hemispheres. Following the manual selection of templates, we applied the MNE's implementations of FastICA and CORRMAP(*67*). CORRMAP allows to manually select an independent component (IC) for one participant and use the chosen component as a template for selecting similar components in other participants. For that analysis, we included the data obtained from the selected components ("bad" data). We computed wPLI scores for each of the three types of artefactual components in each of the face-to-face paradigms and compared between the experimental conditions.

We compared between the mother-infant free interaction and the stranger-infant free interaction (in the Blank condition), we utilized a non-parametric permutation test with mass-univariate ANOVA to find effects associated with the interacting figure (mother vs stranger) on the inter-brain synchrony scores that were computed using the "rejected" components. Results revealed no significant main effect of the interacting figure condition using **(A)** eye movement components ($F_{max(1,46)} = 3.6$, $p = 1$), **(B)** facial muscles components ($F_{max\,(1,46)} = 1.2$, $p = 1$) and **(C)** non – physiological components ($F_{max(1,46)} = 5.2$, $p = 0.2$), indicating no significant differences in neural connectivity between these two face-to-face conditions using the data obtained from the artefactual components of all three types. **(D)** connectivity scores of the clean data following removal of all detected components reported in this analysis. Results revealed a significant main effect of condition ($F_{(1,46)} = 10.8$, $p = 0.047$). Similar to the reported results in our manuscript, this effect indicated higher connectivity in mother-infant condition relative to stranger-infant between the right central area of the Stranger and the right occipito-temporal area of the infant (Mother-Infant: 0.125 ± 0.07, Stranger-Infant: 0.085 ± 0.04, $p = 0.0019$). This analysis supports our findings even following removal of additional components. Next, we compared the BO and the Blank conditions, we utilized a non-parametric permutation test with mass-univariate ANOVA to find effects associated with the Odor (maternal body odor vs a control odor) on the inter-brain synchrony scores that were computed using the "rejected" components. Results revealed no significant main effect of Odor condition using **(E)** eye movement components ($F_{max(1,50)} = 1.98$, $p = 1$), **(F)** facial muscles components ($F_{max\,(1,50)} = 4.4$, $p = 0.44$) and **(G)** non – physiological components ($F_{max(1,50)} = 3.47$, $p = 1$), indicating no significant differences in neural connectivity between BO and Blank conditions using the data obtained from the rejected components of all three types. These results suggest that the reported differences in neural synchrony between experimental conditions are not driven by artifacts.

(H) connectivity scores using the clean data following removal of all detected components reported in this analysis. Our primary analysis utilized a non-parametric permutation test to find effects associated with the Odor (maternal body odor vs a control odor) on the inter-brain synchrony during social interaction with stranger. Results revealed a significant main effect of Odor condition ($F_{(1,50)} = 16.4$, $p = 0.004$). Same as the reported results in our manuscript, this effect indicated higher connectivity in BO condition relative to Blank between the right central area of the Stranger and the right occipito-temporal area of the infant (BO: 0.115 ± 0.05, Blank: 0.082 ± 0.04, $p = 0.00018$). This analysis supports our finding even following removal of additional components.

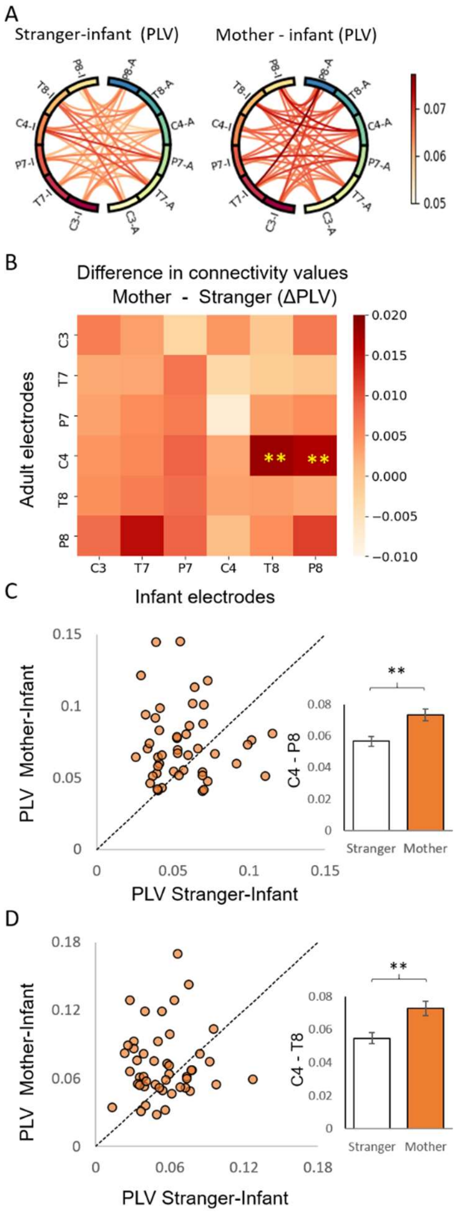

Fig S9.

Phase locking value (PLV) resulted with a similar result to wPLI in the mother-infant comparison. To further validate our main findings, we also used PLV(*24*) to estimate the amount of synchrony between each two electrodes in both mother-infant and stranger-infant conditions. **(A)** Visualization of PLV values in the infant-stranger (left) and the infant-mother free interaction (right) conditions. Each circle represents mean connectivity values for 36 combinations obtained from 47 infant-adult dyads **(B)** Difference in connectivity values across all electrode combinations between the infant-stranger and infant-mother conditions. A non-parametric permutation test with mass-univariate ANOVA revealed a significant main effect of interacting figure (mother vs stranger; $F_{(1,46)}$ = 11.2, p = 0.043) . This effect indicated higher connectivity in the mother-infant condition compared with the stranger-infant condition **(C)** between the right central area of the adult and the right occipito-temporal area of the infant (Stranger: 0.057 ± 0.021, Mother: 0.073 ± 0.026) and **(D)** between the right central area of the adult and the right temporal area of the infant (Stranger: 0.055 ± 0.023, Mother: 0.073 ± 0.030)

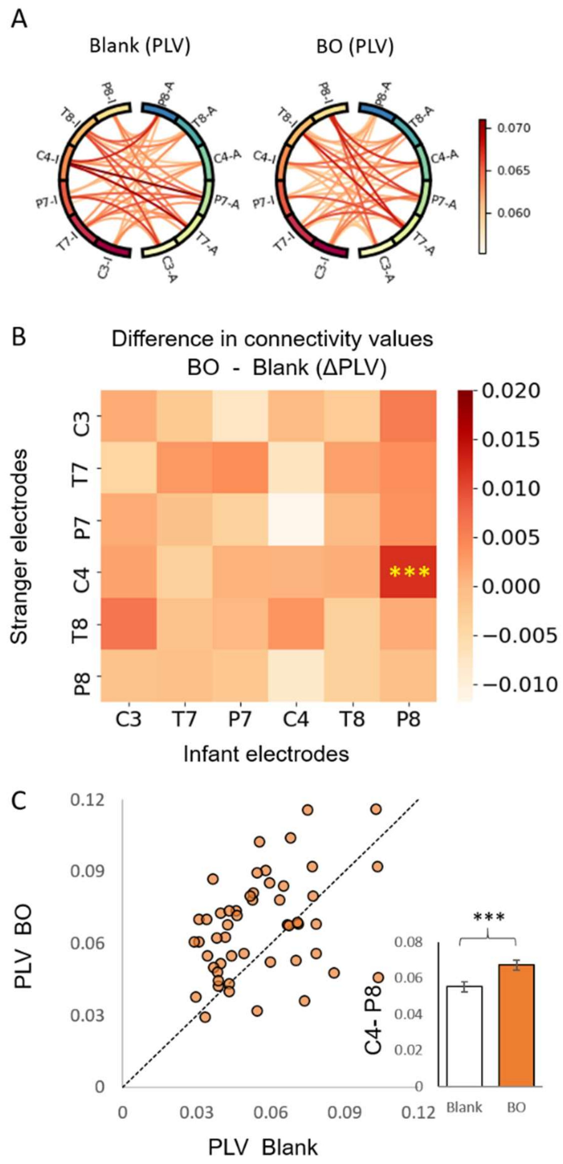

Fig S10.

Phase locking value (PLV) resulted with a similar result to wPLI in the BO-Blank comparison. To further validate our main findings, we also used PLV(*24*) to estimate the amount of synchrony between each two electrodes in both odor conditions. **(A)**. Visualization of connectivity values (PLV) in the blank (left) and the body-odor (BO) (right) conditions. Each circle represents mean connectivity values for 36 combinations obtained from 51 infant-stranger dyads. **(B)** Difference in connectivity values of all electrode combinations between the BO and Blank condition. Our primary analysis utilized a non-parametric permutation test with mass-univariate ANOVA to find effects associated with the Odor (maternal body odor vs a control odor) on the inter-brain synchrony during social interaction with stranger. Results revealed a significant main effect of Odor condition ($F_{(1,50)} = 14.0$, $p = 0.01$). **(C)**This effect indicated higher connectivity in BO condition relative to Blank between the right central area of the Stranger and the right occipito-temporal area of the infant (BO: 0.067 ± 0.02, Blank: 0.055 ± 0.02).

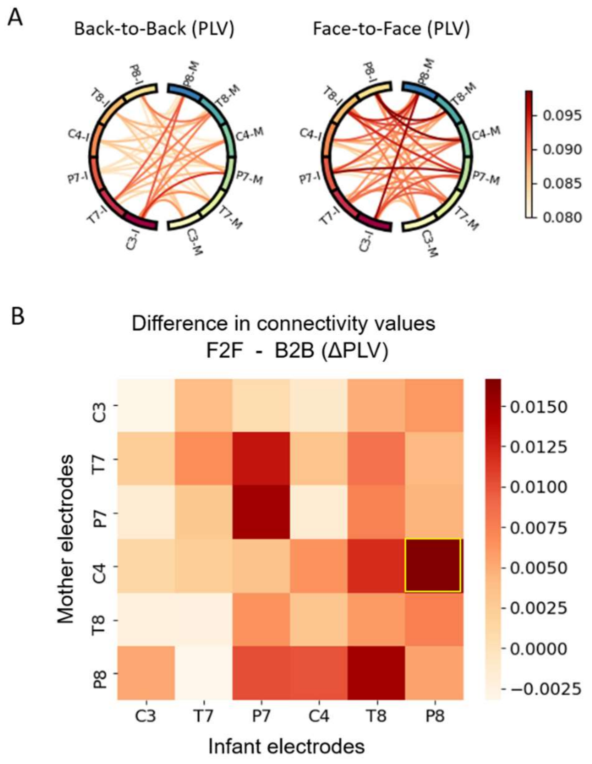

Fig S11.

Phase locking value (PLV) resulted with a similar pattern to wPLI in the F2F-B2B comparison. To further validate our main findings, we also used PLV(*24*) to estimate the amount of synchrony between each two electrodes in both mother-infant conditions. **(A)**. Visualization of connectivity values (PLV) in the Back-to-Back (left) and the Face-to-Face (right) conditions. Each circle represents mean connectivity values for 36 combinations obtained from 37 infant-mother dyads. **(B)** Difference in connectivity values of all electrode combinations between the Face-to-Face and Back-to-Back condition. Our primary analysis utilized a non-parametric permutation test with mass-univariate ANOVA. Although no significant comparisons were detected using PLV, this analysis revealed a similar pattern to the results described with the wPLI. As can be seen, the largest difference between conditions indicates higher connectivity in the Face-to-Face condition relative to Back-to-Back condition between the right central area of the Mother and the right occipito-temporal area of the infant is shown (F2F = 0.99 ± 0.047, B2B = 0.082 ± 0.03) (marked with yellow square).During the Back-to-Back condition both the infant and the mother were watching soap bubbles machine simultaneously. We suspect that the shared visual stimuli may have induced zero-lag synchronization that possibly leads to spurious hyper-connections when using the PLV analysis. These possible spurious hyper-connections in response to external stimuli may mask genuine differences between conditions and was among the main reasons for our choice to analyze the data with wPLI.

Table S1. Sixty-five dyads participated in the study

	Mother Age (years)	Infant Age (months)	Gender	Bubbles	Mother Free Interaction	BO Free Interaction	Blank Free Interaction
Subj01	35.0	11.1	F		1		
Subj02	34.0	8.5	M		1		
Subj03	27.0	7.1	M	1	2		
Subj04	29.0	3.8	M	1	2		
Subj05	34.0	7.1	F		1		
Subj06	34.0	11.4	M		1		
Subj07	31.0	7.5	M		2		
Subj08	32.0	6.9	M	1	2		
Subj09	30.0	7.6	F	1	2	3	4
Subj10	34.0	12.0	F		1	3	2
Subj11	22.0	5.5	F	1	2	4	3
Subj12	27.0	8.6	M		1	2	3
Subj13	42.0	7.6	M	1	2	4	3
Subj14	42.0	6.7	F	1	2	3	4
Subj15	34.0	7.2	M		1	3	2
Subj16	34.0	5.9	M	1	2	4	3
Subj17	28.0	6.4	F	1	2	3	4
Subj18	35.0	7.8	M	1	2		
Subj19	34.0	8.1	M	1	2	4	3
Subj20	30.0	7.8	F	1	2	4	3
Subj21	27.0	8.9	F	1	2	3	4
Subj22	37.0	9.0	M			1	2
Subj23	37.0	8.0	M	1	2	4	3
Subj24	34.0	6.9	F	1	2	3	4
Subj25	37.0	7.3	M		2	4	3
Subj26	34.0	6.8	M	1	2		
Subj27	37.0	7.5	M		1		
Subj28	30.0	8.4	M	1	2		
Subj29	33.0	6.1	F	3	4	1	2
Subj30	35.0	6.8	M	3	4	1	2
Subj31	25.0	6.4	M	3	4	1	2
Subj32	34.0	7.7	F	3	4	2	1
Subj33	35.0	6.2	F	3	4	1	2
Subj34	38.0	6.8	F		3	1	2
Subj35	33.0	7.7	M	3	4	1	2
Subj36	40.0	6.4	F	3	4	2	1
Subj37	38.0	6.8	F	1	2	3	4
Subj38	29.0	6.4	M	4	3	1	2
Subj39	37.0	6.3	F		3	2	1
Subj40	32.0	8.6	F	3	4	2	1
Subj41	30.0	6.1	M	3	4	1	2
Subj42	32.0	7.0	M		3	2	1
Subj43	32.0	7.7	M	3	4	2	1
Subj44	34.0	6.5	F	3	4	2	1
Subj45	35.0	6.9	M	1	2	3	4
Subj46	35.0	6.5	M			1	2
Subj47	25.0	6.3	M	3	4	1	2
Subj48	28.0	6.0	F		3	2	1
Subj49	35.0	4.8	M	2	1	4	3
Subj50	33.0	7.0	M	3	4	2	1
Subj51	36.0	4.9	M	1	2	4	3
Subj52	35.0	5.8	F		1	2	3
Subj53	30.0	5.4	F		1	2	3
Subj54	33.0	6.0	F	1	4	2	3
Subj55	35.0	4.7	F		1	2	3
Subj56	32.0	8.5	M	2	1	4	3
Subj57	32.0	7.3	F	1	2	4	3
Subj58	37.0	5.0	M	1	2	3	4
Subj59	37.0	5.7	M		1		
Subj60	37.0	5.1	M		1	3	2
Subj61	32.0	5.8	F		1	3	2
Subj62	40.0	5.5	F		1	2	3
Subj63	35.0	6.3	M		1	3	2
Subj64	37.0	7.8	F		1		
Subj65	39.0	6.5	M			1	2

The table details the gender and age of all infant participants, as well as their participation in the different paradigms and their order for each participant
